# Self-care for people with heart failure: the importance of tele-nursing in the COVID-19 pandemic*

**DOI:** 10.1590/1518-8345.6975.4227

**Published:** 2024-08-12

**Authors:** Micaelle Costa Gondim, Ricardo Costa da Silva, Ana Karoline Barbosa da Silva, Flaviana Vely Mendonça Vieira, Janaína Valadares Guimarães, Karina Machado Siqueira, Agueda Maria Ruiz Zimmer Cavalcante

**Affiliations:** 1 Universidade Federal de Goiás, Faculdade de Enfermagem, Goiânia, GO, Brazil; 2 Scholarship holder at the Coordenação de Aperfeiçoamento de Pessoal de Nível Superior (CAPES), Brazil; 3 Universidade Estadual de Goiás, Ceres, GO, Brazil

**Keywords:** Chronic Disease, Self-Management, Qualitative Research, Heart Failure, COVID-19, Telemonitoring

## Abstract

**Objective:**

to describe factors of influence of telenursing on naturalistic decision making about self-care of people with heart failure during COVID-19.

**Method:**

this is a descriptive study with a qualitative approach, with 16 participants interviewed after hospitalization for decompensated heart failure. The data was collected using audio and video technology and submitted to content analysis, guided by the Situation-Specific Theory of Heart Failure Self-Care.

**Result:**

two thematic categories emerged from the analysis: Self-care as a decision-making process and Factors influencing decision-making, which describe tele-nursing as a support resource for people with heart failure, during changes in care in the pandemic period.

**Conclusion:**

it was possible to understand the relationship between telenursing and the establishment of a bond, in order to stimulate naturalistic decision-making by people with heart failure in their daily lives in a coherent way, leading to better health outcomes.

## Highlights

**(1)** Different barriers compromise the decision-making of people with heart failure.

**(2)** Nursing theories can guide coherent self-care attitudes.

**(3)** Continuity of care and a support network are necessary after hospitalization.

**(4)** Emotional support for people with heart failure must go hand in hand with conventional treatment.

**(5)** The bond through tele-nursing provides positive health outcomes.

## Introduction

Of the various aspects in which we could analyze the context of the global health crisis, we have as an object of observation for this study people with heart failure (HF), given that they belong to the group of chronic diseases, considered conditions of greater vulnerability to the new type of Coronavirus, Severe Acute Respiratory Syndrome Coronavirus 2 - SARS-COV 2, which causes the disease COVID-19 as a result of acute respiratory syndrome^1^.

As a chronic disease, HF results from important physiological, structural and functional changes, associated with the presence of multimorbidities, high rates of rehospitalization^2^ and the coexistence of multiple emotional symptoms that contribute significantly to cardiovascular decompensation events^3^.

However, due to the COVID-19 pandemic, there has been a significant change in health care on a global scale, both at the outpatient and hospital level, causing a reduction in the monitoring of this population, and consequently, in the number of hospitalizations^4^.

In 2020, there were more than 114,000 hospitalizations for HF across the country, with 11,548 admissions (and readmissions) in the Midwest region, the site of this study^5^. A significant figure, however, with a 15% reduction in hospitalizations compared to the previous year^4^. This lack of follow-up can influence self-care, leading to cardiac decompensation^6^.

The Situation-Specific Theory of HF Self-Care provides nurses with the tools to provide nursing care based on encouraging important changes in lifestyle, the perception and monitoring of symptoms and the achievement of autonomy to carry out self-care through a naturalistic decision-making process (NDT)^7^ ^), (^ ^8^ ^), (^ ^9^.

People with chronic illnesses have significant demands during the course of the disease and need adjustments to their self-care routine^10^. The bond comprises the responsibility between the health team and the individual in promoting health and preventing illness through effective communication, building trust and affective relationships^11^.

When this link is maintained, for example, with telemonitoring, it reinforces the potential of this technological tool to provide care at a distance, facilitate access to health professionals, promote clinical practice, individualized care, clarification of doubts, fears and anxieties, favoring knowledge of the different realities of the people assisted^12^ ^), (^ ^13^.

We consider it important to address the topic, because in addition to the particularities involved in coping with the pandemic during the period of social restrictions, there are important gaps in the literature on the self-care of people with HF based on theories of practice that address limitations and barriers encountered in experiencing the disease. Through the experiences reported after hospitalization, the research sought to describe the influencing factors of telenursing in naturalistic decision-making for self-care of people with HF during COVID-19.

## Method

### Study design

Qualitative study^14^, based on the situation-specific theory of heart failure self-care, which proposes the influence of knowledge, experiences, skills and compatibility with values on the self-care of people with HF^7^.

The design and execution of the research was guided by the recommendations of the Consolidated Criteria for Reporting Qualitative Research (COREQ): a 32-item checklist for interviews and focus groups^15^, using a checklist provided by the EQUATOR network (https://www.equator-network.org/).

### Research scenario

The study was carried out with people linked to a public tertiary hospital institution of the Unified Health System (SUS), which provides outpatient and inpatient cardiology care, in a capital city in the central-western region of the country. The interviews took place after hospitalization as a result of decompensation of the disease, using telenursing consultation as a strategy^16^ ^), (^ ^17^.

### Period

The interviews were conducted between November 2020 and January 2021.

### Study participants and selection criteria

People aged 18 or over diagnosed with HF participated in the study, with the cause of hospitalization recorded in their medical records. Participants were selected from a variety of samples, defined by the criterion of fundamental homogeneity, whose key characteristic for the study was having at least one hospital readmission within six months, due to decompensation of HF^18^.

The search for patients was carried out by listing the monthly admissions by specialty of care and reason for admission of all those who had been readmitted within six months of discharge.

Initially, 48 patients were selected, of whom 22 could not be contacted because their records were out of date. After an active search by telephone, 26 patients were contacted and nine patients were excluded: refusal to participate due to physical weakness (2), death (1), not meeting the eligibility criteria (1), no response to subsequent telephone contact (5). One patient was considered lost after the start of the interview due to a drop in the internet connection, without success in a new connection to continue the interview, even after three attempts, on alternate days. A total of 16 participants were included.

The exclusion criteria were people who had suffered a stroke with cognitive sequelae, pregnant women, patients with congenital HF, patients who had undergone major surgery three months before data collection, deafness and cognitive limitations that made communication difficult.

### Data collection

Once the list of patients eligible for the study had been established, an invitation letter was sent via the WhatsApp^®^ instant messaging platform, reinforcing the purpose of the study, introducing the researcher and the reasons for carrying out the study.

Due to the social restrictions caused by the COVID-19 pandemic, all contact with the participants took place via the patient’s or family member’s own messaging app, and via telephone call. Data collection was carried out by a nurse researcher with training and experience in conducting qualitative studies, in a single moment, on a date and time indicated by the participant. It was made clear that the interview would be prioritized via video call, but due to the choice of some participants (N=6), the interview was conducted via audio call.

The reading of the informed consent form was recorded in full, as was the entire interview after acceptance, using a mobile device and available through the iOS operating system for iPhone^®^. The interviews lasted an average of 34 minutes (18 minutes minimum and 60 minutes maximum).

The data collection instrument consisted of a semi-structured script that allowed participants to talk about their experience with the disease, the self-care measures they adopted on a daily basis, their perceptions of symptoms and how to deal with signs of decompensation, as well as any information they might have received during hospitalization about self-care.

The 5 questions were designed in accordance with the theory underpinning the study^7^ ^), (^ ^8^ ^), (^ ^9^: 1) Can you tell me what it’s like to live with HF?; 2) Tell me what you do on a daily basis to take care of yourself and your HF; 3) When you notice that something isn’t right, what actions do you take that you think could help solve the problem?; 4) What do you think may have contributed to the occurrence of more than one hospitalization during this year?; 5) Do you have any examples of information you received on how to take better care of yourself and your illness during hospitalization?

Sociodemographic and clinical data were collected from the medical records and checked with the participants at the start of the interview.

After data collection, due to the pandemic period, which led to difficulties in accessing the health service, many doubts and questions were raised by the participants about appropriate conduct in managing the disease and issues inherent to the health service. Therefore, due to the ethical commitment of the researcher responsible for data collection, the requested information was provided at the end of the interview.

### Data analysis

The content analysis technique was used, using the thematic modality^19^. The trail-based analytical procedure was anchored in the theoretical framework of the Situation-Specific Theory of Self-Care in Heart Failure^8^. Content analysis consists of a set of systematic techniques and procedures for understanding the content of interviews in order to infer relevant knowledge. Floating reading of the interview after transcription was followed by a thorough reading with the establishment of units of meaning, subcategories and, finally, the categories that represented the refinement of the results^19^.

The proposed thematic analysis was achieved through in-depth reading and, in order to guarantee the principle of plausibility of the data collected and the interpretation of the research results, the analysis was carried out by two researchers and the ways of identifying the categories were discussed, seeking consensus on the themes identified^18^.

In order to systematize the analysis process and manage the textual material, the data was processed using ATLAS.ti^®^ software version 9.0. Speeches transcribed in a Microsoft Word document were sent to the software for the creation of units of meaning or codes, which were found in significant numbers (N=370). This technological resource promoted greater scientific rigor in grouping the codes to form subcategories, from which the following themes emerged.

During this process, the subcategories and themes were analyzed in the light of the Situation-Specific Theory of HF Self-Care, in order to identify whether they represented the dimensions described in the autonomous and consultative elements of the theory.

The participants were identified with the code P followed by the number assigned to them in the order of the interview (P1, P2, P3, (...), P16).

### Ethical aspects

The study followed the ethical precepts of research involving human beings, according to Resolution 466/2012 of the National Health Council, and was approved by the participating institution under opinion number 4.161.021, CAEE No. 10225519.8.0000.5078, version 3.

After the interviews, in accordance with the ethical commitment of the research, the participants were sent an information leaflet prepared by the researchers on self-care for HF, based on the stages of the situation-specific theory of self-care for HF and the assumptions of the middle-range theory of chronic diseases^7^ ^), (^ ^8^ ^), (^ ^9^ ^), (^ ^20^.

## Results

According to the characterization of the study participants (N=16), the majority were male (N=13), aged between 37 and 85, especially over 65 (N=9), married (N=10), with a diagnosis time of more than 5 years (N=12). The classification of HF was collected from the medical records. The hemodynamic clinical profile was predominant, with a prevalence of Profile B (N=10).

The profile of the participants reflected high cardiac involvement, with obvious symptoms and signs of edema and congestion, as well as associated comorbidities such as arterial hypertension, renal alteration and/or diabetes.

Considering that the inclusion criterion for the study was to have undergone more than one hospitalization, a considerable number of re-hospitalizations were observed in the period analyzed. Self-reported hospitalizations ranged from two to eight, with eight participants reporting three hospitalizations during the year and five having undergone more than four hospitalizations within a six-month interval.

It was clear from the reports that the hospital is indeed the reference for treating the disease. However, the changes in care due to the pandemic and the consequences suffered by this population are reflected in the barriers imposed by the health service.

**Figure 1:**
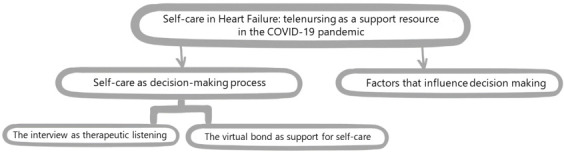
Categories and subcategories of the qualitative interview of the study “Self-care of people with heart failure: the importance of telenursing in the COVID-19 pandemic”. Brazil, 2023

In-depth reading and analysis of the material derived from the interviews generated two thematic categories that are close to the naturalistic decision-making process (NDM) for carrying out self-care^7^ ^), (^ ^8^ ^), (^ ^9^, highlighting the barriers and limitations identified in the statements, as shown in Figure 1.

**Figure 2:**
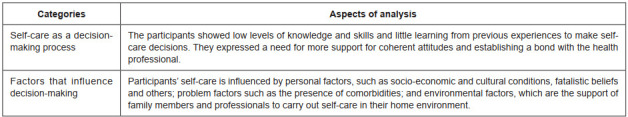
Aspects of analysis of the categories Self-care as a decision-making process and Factors influencing decision-making. Brazil, 2023

Figure 2 shows the categories and aspects of analysis.

### Self-care as a decision-making process

This category includes the aspects that influence participants’ daily self-care decisions: knowledge, experience, skills and values^7^ ^), (^ ^8^ ^), (^ ^9^. All of these aspects were identified quite poorly and at levels far below what was expected, given the length of the diagnosis reported. Two subcategories emerged from this category: The interview as therapeutic listening and The virtual bond as support for self-care.

In the subcategory The interview as therapeutic listening, we highlight speech strata that show the relevance of the moment of the interview through reports of family problems, expression of the feelings present when living with HF; such as feelings of powerlessness, expression of faith, worries and anguish:

*[...] I liked working [...] I miss it a lot (emotional) [...] I’m also very Catholic, and that helps me a lot [...]* (P1).

*[...] my wife died recently and we have a three-year-old child, it’s very difficult because I can’t work [...] I still have a lot of problems (emotional) [...]* (P9).

*[...] my life has been very difficult for me, very distressing [...] I try to laugh, to live things in grace, because I live with God [...] it gets so painful, it gets so bad, you know? it’s very difficult, I don’t even know what I’m going to do, honestly [...] I used to think about controlling it with food, right?* (P11)

*[...] but this year has been the worst year of my life, since January [...] I’ve been hospitalized 6 times* (P12).

*[...] When my heart fails, I don’t do anything, then it passes, then I walk, I try to forget about it [...] the doctor told me that it’s a bit of my head, because I get caught up in it, so I try to forget about it [...]* (P4).

*It’s too tiring. And you worry that you don’t know if you’re doing the right thing or the wrong thing [...]* (P13).

Parts of speech were highlighted in the subcategory virtual bond as support for self-care, expressing requests for help with issues inherent to the disease and/or specific bureaucratic procedures. During the interview, it was clear that there were important needs for adjustments to control the disease, which are essential, and that there was a lack of professionals who could provide this support. It is clear that the data collection reached the configuration of a nursing consultation and reflected the shortcomings of the hospitalization period for the learning of people with HF. Furthermore, the relevance of tele-nursing in monitoring patients at home was noted:

*[...] is it real or are they fooling us (referring to the blood pressure values on the portable device)? (...) But it’s difficult. I’d like to win it (referring to the medication). How do I get it?* (P8).

*I’d like to find out if there’s any danger of me falling at some point and dying because of this disease* (P10).

*I’ll book your phone number straight away, and if I have any questions, can I ask you?* (P12).

*[...] now when it comes to my heart, when I get sick [...] I go to hospital [...] I don’t even use diuretics because I know they don’t help, only in hospital [...] do you know how to help me get a medical certificate so I can retire?* (P13).

*[...] my wife also has a heart problem, some days her blood pressure is very high, can you talk to her? (handing the phone to his wife)* (P15).

### Factors that influence decision-making

The speech strata of the Factors influencing decision-making category represent the factors highlighted in the situation-specific theory of HF self-care^8^: personal factors such as socio-economic, cultural and spiritual conditions, health literacy and the presence of fatalistic beliefs; problem factors such as the presence of more than one comorbidity, reported by almost all of the study participants (N=13); and environmental factors related to the support received, both from professionals and family members, to carry out self-care in their family environment. It also includes emotional support to modulate the stress involved in managing the disease and the way the person deals with their emotions:

*[...] I wasn’t feeling well, you know? then she didn’t give me much credit (doctor) [...] the emergency room was full, there was no room [...] with this pandemic they asked me to stay at home, but here at home I was having a really bad time* (P5).

*[...] there are days when I don’t sleep, I spend the whole night praying [...] I get very ill, I really do, only the bed makes it better. I stay in bed for one, two, three or even four days to get better* (P7).

*[...] today I do almost nothing standing up, I have to do everything sitting down [...] I look after myself [...]* (P8).

*My wife remembers the medication, I can’t manage* (P10).

*[...] I get sick at night sometimes, I call him (the doctor), or my wife, and he gives me advice. He’s an angel of God to me [...] I also have a lot of faith in home remedies* (P12).

*There are programs (cites name of hospital), but because of the pandemic they have been suspended. I’m waiting for them to come back, like cardiovascular rehabilitation [...] you gave me very good attention [...]* (P13).

*When I was in the program (participating in another study carried out at the hospital), I used to call the secretary (of the doctor) now I’m not, I don’t call anymore [...] my daughter is a nurse, so even if I don’t think I have to go, she makes me go (to the hospital)* (P16).

## Discussion

It is clear from the participants’ speeches that there is little outpatient care, especially in more complex situations. The testimonies point mainly to the abandonment of physical activities, carried out at the institution’s physiotherapy outpatient clinic and/or spontaneously in public leisure areas, difficulties in receiving care due to crowded emergency units, delays in post-discharge return appointments and in the cardiology outpatient clinic.

When questioned, most of the participants had no contact with health professionals to answer questions and provide guidance on day-to-day conduct, nor had they received guidance on how to take better care of themselves during hospitalization. When guidance was given, it was more related to the routine of the nutrition service offered to all patients, without individualizing their needs. However, through reports of gratitude for the care received and the relationship with the healthcare team, the presence of the medical professional was emphasized, with the nurse, as well as other professionals from the multi-professional team, being barely mentioned in the speeches.

A multicenter study tested HF management programs in public and private institutions in Brazil and warned about the gap in patient and caregiver education, and the underuse of patient monitoring (home visits and/or telemonitoring) in both types of services^21^. The low effectiveness of the information provided to reduce negative results in the treatment of HF was related to both pharmacological and non-pharmacological treatment.

There are also gaps in knowledge about the pathology, the disease process, as well as important non-pharmacological measures for adopting self-care behaviors that promote a better quality of life. Where they did exist, there were incipient reports of attempts at self-care attitudes such as sodium control, reducing fluid intake, frequent physical activity and monitoring symptoms of decompensation. Pharmacological treatment was the most commonly mentioned, but adherence was poor.

Many participants reported doubts about adjustments to their diet and medication and the search for answers about their clinical condition to allay fears and concerns. Others expressed doubts about bureaucratic procedures at the hospital of reference, showing a desire for a greater bond with the professionals.

In the presence of chronic illnesses, self-care is essential for maintaining health, requiring a set of behaviors that lead to health promotion and disease management^20^. HF guidelines emphasize the importance of education in patient adherence to treatment, lifestyle changes, monitoring symptoms and responding appropriately to possible signs of disease decompensation^2^ ^), (^ ^22^.

The situation-specific theory of HF self-care defines self-care as a naturalistic decision-making process, divided into linear stages to be completed by the person with HF: 1) Maintenance of self-care, which involves lifestyle adjustments, 2) Symptom perception, including monitoring and the body reading process, and 3) Self-care management, with the acquisition of skills and the adoption of behaviors in the event of alterations^7^ ^), (^ ^8^ ^), (^ ^9^.

Each stage involves consultative elements, where the person receives help and support on what to do and develops autonomy from acquiring the knowledge and confidence to look after themselves^7^ ^), (^ ^8^ ^), (^ ^9^. In the light of the situation-specific theory of HF self-care, people make decisions automatically, incompletely, contextually and rarely following a model. However, it can be influenced by various factors, such as: emotional support, support received, experience, knowledge, cultural, values and others^9^.

The important role of the informal caregiver should also be considered, as well as professional support that enables continuity of care and avoids re-hospitalizations as the only way to solve the problem^23^ ^), (^ ^24^ ^), (^ ^25^ ^), (^ ^26^.

Since the COVID-19 pandemic, nursing teleconsultation has been regulated by COFEN Resolution No. 634/2020, which “authorizes and regulates nursing teleconsultation through consultations, clarifications, referrals and guidance using technological means”^27^. Therefore, nurses must use their knowledge, skills and other available resources to provide people with more comprehensive and effective care^28^ ^), (^ ^29^.

Self-care involves multiple factors that are susceptible to intervention. It is a process subject to influences that can favor better choices and result in better outcomes^30^. Since the 1980s, research into naturalistic decision-making has sought answers to how people make decisions in the real world^31^.

The limited number of reports in our study about information received during hospitalization and the lack of knowledge about the factors that influence the decompensation of the disease hinder the process of self-care and decision-making. The participants did not show that they were prepared to make the necessary decisions in situations of decompensation.

According to the NDM theory, people make choices based on rational and/or intuitive components, and when they make their choices, they rarely do so in an analytical and systematic way. In other words, people make decisions for various reasons, but there is a consensus that their experiences trigger quick choices in critical situations so that they commit to a course of action, without necessarily comparing the other existing alternatives^31^ ^), (^ ^32^.

In many of the interviews, they vented their experiences of receiving the diagnosis, mentioning personal values, the impact of lifestyle changes, the lack of motivation to take care of themselves, reports of traumatic situations that influence adherence to treatment and the acquisition of autonomy to manage self-care. This is evidence of the participants’ need to be heard in their anguish and anxieties.

The participants in the study seem to have insufficient knowledge, even with considerable time since their diagnosis; when they do have it, few report the confidence to adopt coherent attitudes. An example of this is the lack of autonomy to take extra diuretics in the presence of edema.

According to concepts described in the middle-range theory of self-care for chronic diseases, motivation can be intrinsic or extrinsic, with the former arising from the desire to learn and perform tasks that generate pleasure or satisfaction, and the extrinsic arising from understanding the need to improve health, or even aiming to please other people, such as family members^20^.

Many self-care behaviors that arise from extrinsic motivation evolve into intrinsic motivation, shaping a lifestyle that is pleasurable for the person and with greater chances of success^20^. This explanation is related to the understanding of decision-making based on combinations of learned patterns, a purely intuitive factor. If the person makes a clear correspondence between their choices and the results obtained, they will be able to take actions that are more coherent with their health condition and needs and involve more assertive choices based on reasoning in the face of different existing situations^31^ ^), (^ ^33^.

Cultural beliefs and values were also very evident, such as the high level of dependence on the spouse, religious influences, popular beliefs in home remedies and other behaviors that can be worrying, contradicting recommendations based on scientific evidence.

Due to the lack of follow-up, the emotional overload of the participants in this study was evident. The explicit or hidden needs in many moments of speech reflect the need to have someone who was really interested in listening to their testimonies, life and daily stories, frustrations, difficulties, anxieties, desires, or even someone who could provide psychological support in coping with the disease.

Psychological conditions can contribute more negatively to the worsening of the disease than physiological causes, due to the impact on people’s lives, the lack of follow-up and/or inadequate symptom management^34^.

In this sense, the researcher occupies an important place in human relationships of subjective understanding of the other person’s experience, regardless of whether the information makes sense to the researcher themselves^35^. In qualitative research, the participant and the researcher are interconnected parties in the investigation, where understanding the phenomenon depends on an empathetic, sensitive and unique movement^14^. The meanings of conversations depend on the context in which they occur^15^.

We attribute the depth of the themes that emerged during the interview to the qualitative approach which, although an interview script with semi-structured questions was used, allowed for this moment of empathy with the researcher, therapeutic listening and a favorable environment for the participants to expose their reality and feelings.

This therapeutic listening is understood as an essential strategy for understanding the other, capable of defining an order of priority for assistance based on the individual needs expressed by people and capable of sending health professionals on ways to improve people’s decision-making performance^31^ ^), (^ ^36^.

A phenomenological study on social distancing during the pandemic highlights that the impossibility of interacting in person has had a profound impact on social relationships^37^, while “interaction with another person can nurture a sense of comfort and hope or, on the contrary, a sense of discomfort and vulnerability”^37^. It is necessary to consider the context of interpersonal interactions shared by the other person and the other person’s existing “being in the world”, in which we are invited to participate, and which makes the depth of interpretation and the helping process possible.

Still largely unknown, these virtual experiences must be analyzed to bring greater clarity to the dimensions of this new era, in the hope of getting positive responses to a new way of assisting others. Our data also strengthens our understanding of the need for comprehensive interventions and nursing care that is more accessible to people in their home environment, where their greatest doubts and difficulties arise, their role in the family nucleus and their identity as a social being.

The confidence mentioned above, as a fundamental element for managing self-care, reflects people’s self-efficacy, i.e. the ability to make coherent decisions even in the face of barriers and difficulties, and is not part of self-care itself, but is essential at all stages^7^ ^), (^ ^8^ ^), (^ ^9^. The need for support and encouragement for the study participants is evident.

**Figure 3:**
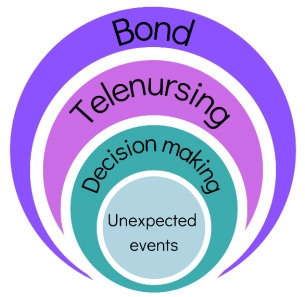
Diagram representing the relationship between the bond with people with heart failure, the influence of telenursing and the naturalistic decision-making process for self-care in situations of unexpected events. Brazil, 2023

Analysis of the interviews leads us to realize that in the event of an unexpected event (extrinsic, such as the pandemic or another stressful situation in everyday life, or intrinsic, such as decompensation of HF and exacerbation of symptoms) it is important for the person to be able to carry out the decision-making process for self-care, in this sense telenursing can be a collaborative resource in the management of these events, in strengthening the bond previously established, or in the possibility of establishing this bond in the post-hospitalization period. To represent this interrelationship, a diagram was drawn up as shown in Figure 3.

According to the National Primary Care Policy (PNAB), the bond “consists of building relationships of affection and trust between the user and the health worker, allowing for a deepening of the process of co-responsibility for health, built up over time, as well as carrying therapeutic potential” (p. 21)^38^.

Through telenursing, the establishment of a bond can begin with the acquisition of information that enables interventions even at a distance, lifestyle guidelines, and clarification of doubts to prevent early readmission^39^.

A study carried out by nurses trained in tele-coaching in Germany during the COVID-19 pandemic, who assisted people with HF in the post-discharge period, showed that the group accompanied by telemonitoring recognized the symptoms of decompensation of the disease and learned about the time to seek medical assistance, avoiding situations of greater severity^40^.

Similarly, in our study, telenursing represented an important tool in this context of global health crisis by promoting empathy, respect, active listening, and individualized, comprehensive therapeutic care for patients with HF.

The study contributes to the advancement of science by demonstrating that the use of tele-nursing can overcome important barriers imposed between people with HF and health services. However, the use of theories of practice can instrumentalize and direct the assistance of nurses and other members of the healthcare team in clarifying doubts and supporting the achievement of the stages of self-care. More qualitative studies are needed to provide new evidence of these relationships.

The limitations of this study were the diversity of the participants’ sociodemographic and clinical factors and the difficulties some participants had in using electronic communication resources, especially video media.

## Conclusion

The study showed, in the light of the situation-specific theory of self-care for heart failure and the middle-range theory of self-care for chronic diseases, the existence of important gaps that compromise the decision-making of people with HF, which have a significant impact on the acquisition of skills for the proper management of self-care.

Considering the pandemic period, this population suffered the negative consequences of the lack of follow-up needed to manage the disease. In view of this, tele-nursing is an important resource for establishing a bond with the patient after a period of hospitalization.

The presence of the nurse was barely noticed in the reports, which is a warning that the lack of an active presence by the professional as an educator result in a lack of knowledge and understanding of these people in the context of health services. This perception was clear in the requests made by the study participants with the doubts presented and the appreciation of this moment of interview.

The need for continuity of care and the establishment of a support network for people after hospitalization was evident. The interview was an integral therapeutic moment in telenursing, given the barriers imposed by the COVID-19 pandemic.

It also highlighted the importance of emotional support for people with HF, combined with conventional treatment, to better cope with the disease. However, it is necessary to reflect on how effective monitoring is for this population in all contexts, even outside the pandemic period.

## References

[1] Bader F, Mania Y, Atallah B, Starling RC (2021). Heart failure and COVID-19. Heart Fail Rev.

[2] Rohde LEP, Montera MW, Bocchi EA, Clausell NO, Albuquerque DC, Rassi S (2018). Diretriz brasileira de insuficiência cardíaca crônica e aguda. Arq Bras Cardiol.

[3] Harris KM, Jacoby DL, Lampert R, Soucier RJ, Matthew BM (2021). Psychological Stress in Heart Failure: A Potentially Actionable Disease Modifier. Heart Fail Rev.

[4] Manzanaresa RG, Rodríguez CP, Fernández IG, Domínguez JCC, Sánchez MA (2020). Hospitalización por insuficiencia cardíaca durante la pandemia de COVID-19. Semergen.

[5] Ministério da Saúde (BR) (2024). Morbidade Hospitalar do SUS - por local de internação - Brasil.

[6] Jaarsma T, Hill L, Bayes-Genis A, La Rocca HPB, Castiello T, Celutkiene J (2021). Self-care of heart failure patients: practical management recommendations from the Heart Failure Association of the European Society of Cardiology. Eur J Heart Fail.

[7] Riegel B, Dickson VV (2008). A Situation-Specific Theory of Heart Failure Self-care. J Cardiovasc Nurs.

[8] Riegel B, Dickson VV, Faulkner KM (2016). The situation-specific theory of heart failure self-care revised and updated. J Cardiovasc Nurs.

[9] Riegel B, Dickson VV, Velone E (2022). The situation specific theory of heart failure -an update on the problem, person, and environmental factors influencing heart failure self-care. J Cardiovasc Nurs.

[10] Werner NE, Tong M, Borkenhagen A, Holden RJ (2019). Performance-Shaping Factors Affecting Older Adults' Hospital-to-Home Transition Success: A Systems Approach. Gerontologist.

[11] Costa MFBNA, Sichieri K, Poveda VB, Baptista CMC, Aguado PC (2020). Transitional care from hospital to home for older people: implementation of best practices. Rev Bras Enferm.

[12] Haynes SC, Tancredi DJ, Tong K, Hoch JS, Ong MK, Ganiats TG (2021). The Effect of Rehospitalization and Emergency Department Visits on Subsequent Adherence to Weight Telemonitoring. J Cardiovasc Nurs.

[13] Mizukawa M, Moriyama M, Yamamoto H, Rahman M, Naka M, Kitagawa T (2019). Nurse-led collaborative management using telemonitoring improves quality of life and prevention of rehospitalization in patients with heart failure a pilot study. Int Heart J.

[14] Minayo MCS (2007). O desafio do conhecimento: pesquisa qualitativa em saúde. Cien Saude Colet.

[15] Souza VRS, Marziale MHP, Silva GTR, Nascimento PL (2021). Translation and validation into Brazilian Portuguese and assessment of the COREQ checklist. Acta Paul Enferm.

[16] Fronczek AE (2019). Nursing Theory in Virtual Care. Nurs Sci Q.

[17] Ding H, Jayasena R, Chen SH, Maiorana A, Dowling A, Layland J (2020). The Effects of Telemonitoring on Patient Compliance With Self-Management Recommendations and Outcomes of the Innovative Telemonitoring Enhanced Care Program for Chronic Heart Failure: Randomized Controlled Trial. J Med Internet Res.

[18] Turato ER (2013). Tratado da metodologia da pesquisa clínico-qualitativa: Construção teórico-epistemológica, discussão comparada e aplicação nas áreas da saúde e humanas.

[19] Bardin L (2016). Analise de Conteúdo.

[20] Riegel B, Jaarsma T, Strömberg A (2012). A middle-range theory of self-care of chronic illness. Adv Nurs Sci.

[21] Bocchi EA, Moreira HT, Nakamuta JS, Simões MV, Las Casas AA, Costa AR (2021). Implications for clinical practice from a multicenter survey of heart failure management centers. Clinics.

[22] Heidenreich PA, Bozkurt B, Aguilar D, Allen LA, Byuin JJ, Colvin MM (2022). 2022 AHA/ACC/HFSA Guideline for the Management of Heart Failure: A report of the american College of Cardiology/American Heart Association Joint Commitee on Clinical Practice Guidelines. Circulation.

[23] Lee CS, Freedland KE, Jaarsma T, Strombereg A, Vellone E, Page SD (2023). Patterns of self-care decision-Making and associated factors: A cross-sectional observational study. Int J Nurs Studies.

[24] Sousa JP, Santos M (2019). Symptom Mangement and Hospital Readmission in Heart Failure Patients: A Qualitative Study From Portugal. Crit Care Nurs Q.

[25] Jeremias T, Gomes B, Rosa C, Lavareda S (2020). Transitional care to caregivers of dependent older people an integrative literature review. Rev Bras Enferm.

[26] DeVore AD, Granger BB, Fonarow GC, Al-Khalidi HR, Albert NM, Lewis EF (2021). Effect of a Hospital and Postdischarge Quality Improvement Intervention on Clinical Outcomes and Quality of Care for Patients With Heart Failure With Reduced Ejection Fraction: The CONNECT-HF Randomized Clinical Trial. JAMA.

[27] Conselho Federal de Enfermagem (2020). Resolução COFEN no 634/2020. Autoriza e normatiza, “ad referendum” do Plenário do Cofen, a teleconsulta de enfermagem como forma de combate à pandemia provocada pelo novo coronavírus (Sars-Cov-2), mediante consultas, esclarecimentos, encaminhamentos e orientações com uso de meios tecnológicos, e dá outras providências.

[28] Búrilová P, Pokorná A, Búril J, Kantorová L, Slezaková S, Svobodová Z (2022). Identification of telehealth nursing approaches in the light of the COVID-19 pandemic-A literature review. J Nurs Manag.

[29] Guedes HCS, JNB Silva, Januário DC, Trigueiro DRSG, Leadebal ODCP, Barreto AJR (2023). Tecnologias da informação como apoio organizacional das ações de enfrentamento da COVID-19: discurso de enfermeiros. Rev. Latino-Am. Enfermagem.

[30] Clements L, Frazier SK, Lennie TA, Chung ML, Moser DK (2023). Improvement in Heart Failure Self-Care and Patient Readmissions with Caregiver Education: A Randomized Controlled Trial. Western J Nurs Res.

[31] Klein G (2008). Naturalistic decision making. Hum Factors.

[32] Andreis FA Theoretical Approach to the Effective Decision-Making Process (2020). Open J Appl. Sci.

[33] Müller-Staub M, de Graaf-Waar H, Paans W (2016). An Internationally Consented Standard for Nursing Process-Clinical Decision Support Systems in Electronic Health Records. Comput Inform Nurs.

[34] Davis MCR, Dieckman NF, Hansen L, Gupta N, Hiatt S, Lee C (2023). Are Physical and Depressive Symptoms Different Between Women and Men With Heart Failure? An Exploration Using Two Analytic Techniques. J Cardiovasc Nurs.

[35] Benjumea CC (2003). El Investigador Como Instrumento Flexible de la Indagación. Int J Qual Methods.

[36] Ryan RE, Connolly M, Bradford NK, Henderson S, Herbert A, Schonfeld L (2022). Interventions for interpersonal communication about end of life care between health practitioners and affected people. Cochrane Database Syst Rev.

[37] Carel H, Ratcliffe M, Froese T (2020). Reflecting on experiences of social distancing. Lancet.

[38] Ministério da Saúde (BR) (2017). Portaria nº 2.436, de 21 de setembro de 2017. Aprova a Política Nacional de Atenção Básica, estabelecendo a revisão de diretrizes para a organização da Atenção Básica, no âmbito do Sistema Único de Saúde (SUS).

[39] Berghetti L, Danielle MBA, Winter VDB, Petersen AG, Lorenzini E, Kolankiewicz ACB (2023). Transição do cuidado de pacientes com doenças crônicas e sua relação com as características clínicas e sociodemográficas Rev. Latino-Am. Enfermagem.

[40] Knoll K, Leiter SM, Rosner S, Trenkwalder T, Erben A, Kloss C (2022). Impact of Tele-Coaching During the COVID-19 Pandemic on Risk-Reduction Behavior of Patients with Heart Failure. Telemed J E Health.

