# Supplementary material for: Self-care for people with heart failure: the importance of tele-nursing in the COVID-19 pandemic
Source: Rev Lat Am Enfermagem. 2024 Aug 12;32:e4227. doi: 10.1590/1518-8345.6975.4227 (PMC11321188; doi:10.1590/1518-8345.6975.4227)
Supplement: Translation

# Translation: Autocuidado de pessoas com Insuficiência Cardíaca: a importância da telenfermagem na pandemia da COVID-19*

**DOI:** 10.1590/1518-8345.6975.4228

**Authors:** Micaelle Costa Gondim, Ricardo Costa da Silva, Ana Karoline Barbosa da Silva, Flaviana Vely Mendonça Vieira, Janaína Valadares Guimarães, Karina Machado Siqueira, Agueda Maria Ruiz Zimmer Cavalcante

**Affiliations:** 1 Universidade Federal de Goiás, Faculdade de Enfermagem, Goiânia, GO, Brasil; 2 Bolsista da Coordenação de Aperfeiçoamento de Pessoal de Nível Superior (CAPES), Brasil; 3 Universidade Estadual de Goiás, Ceres, GO, Brasil

**Keywords:** Doença Crônica, Auto Gerenciamento, Pesquisa Qualitativa, Insuficiência Cardíaca, COVID-19, Telemonitoramento

## Abstract

**Objetivo:**

descrever fatores de influência da telenfermagem na tomada de decisão naturalista sobre o autocuidado de pessoas com insuficiência cardíaca durante a COVID-19.

**Método:**

estudo descritivo de abordagem qualitativa, com 16 participantes entrevistados após hospitalização pela descompensação da insuficiência cardíaca. Os dados foram coletados por tecnologia de áudio e vídeo e submetidos à análise de conteúdo, norteada pela Teoria de Situação Específica do Autocuidado da Insuficiência Cardíaca.

**Resultados:**

duas categorias temáticas emergiram da análise: *Autocuidado como processo de tomada de decisão* e *Fatores que influenciam a tomada de decisão*, as quais descrevem a telenfermagem como recurso de apoio à pessoa com insuficiência cardíaca, durante modificações de atendimento no período pandêmico.

**Conclusão:**

foi possível compreender a relação entre a telenfermagem e o estabelecimento de vínculo, de maneira a estimular a tomada de decisão naturalista pela pessoa com insuficiência cardíaca em seu cotidiano de forma coerente, impulsionando melhores resultados em saúde.

## Destaques

**(1)** Diferentes barreiras comprometem a tomada de decisão de pessoas com IC.

**(2)** Teorias de enfermagem podem direcionar atitudes coerentes de autocuidado.

**(3)** Faz-se necessária a continuidade da assistência e rede de apoio após hospitalização.

**(4)** O apoio emocional à pessoa com IC deve estar aliado ao tratamento convencional.

**(5)** O vínculo através da telenfermagem proporciona desfechos positivos em saúde.

## Introdução

Dos diversos aspectos em que poderíamos analisar o contexto de crise sanitária mundial, temos como objeto de observação para este estudo as pessoas com insuficiência cardíaca (IC), haja vista que pertencem ao grupo das doenças crônicas, considerado como uma das condições de maior vulnerabilidade para o novo tipo de Coronavírus, o *Severe Acute Respiratory Syndrome Coronavirus* 2 - SARS-COV 2, causador da doença COVID-19, em decorrência da síndrome respiratória aguda^1^.

Como uma doença crônica, a IC resulta de importantes modificações fisiológicas, estruturais e funcionais, associada à presença de multimorbidades, elevadas taxas de rehospitalização^2^ e coexistência de múltiplos sintomas emocionais que contribuem significativamente para os eventos de descompensação cardiovascular^3^.

Entretanto, devido à pandemia da COVID-19, houve uma alteração importante nos atendimentos de saúde em um cenário mundial, tanto a nível ambulatorial quanto hospitalar, ocasionando redução no acompanhamento a essa população, e consequentemente, no número de internações hospitalares^4^.

No ano de 2020, foram mais de 114 mil internações pela IC em todo país, sendo 11.548 admissões (e readmissões) na região Centro-Oeste, local em que este estudo foi desenvolvido^5^. O número expressivo, contudo, obteve uma redução de 15% das internações em relação ao ano anterior^4^. Nesse sentido, a falta do acompanhamento pode influenciar na realização do autocuidado levando à descompensação cardíaca^6^.

A Teoria da Situação Específica do Autocuidado da IC instrumentaliza enfermeiros para uma assistência de enfermagem pautada no incentivo de mudanças importantes no estilo de vida, na percepção, monitoramento dos sintomas e no alcance de autonomia para realização do autocuidado por meio de um processo de tomada de decisão naturalista (TDN)^7^ ^), (^ ^8^ ^), (^ ^9^.

Pessoas com doenças crônicas apresentam demandas importantes no curso da doença e necessitam de ajustes na rotina para o autocuidado^10^. O vínculo compreende a responsabilização entre a equipe de saúde e o indivíduo na promoção da saúde, prevenção de agravos por meio da efetiva comunicação, construção de confiança e relações de afetividade^11^.

Quando o vínculo é mantido, por exemplo, com o telemonitoramento, reforça-se o potencial dessa ferramenta tecnológica para prestar cuidados à distância, facilitar o acesso ao profissional de saúde, favorecer a prática clínica, o cuidado individualizado, o esclarecimento de dúvidas, medos e anseios, favorecendo o conhecimento de diferentes realidades das pessoas assistidas^12^ ^), (^ ^13^.

Consideramos importante a abordagem do tema, pois, para além das particularidades que envolvem o enfrentamento da pandemia durante o período de restrições sociais, existem lacunas importantes na literatura sobre o autocuidado de pessoas com IC que sejam fundamentadas em teorias da prática que abordem limitações e barreiras encontradas no experienciar da doença. Através das experiências relatadas após hospitalização, a pesquisa buscou descrever os fatores de influência da telenfermagem na tomada de decisão naturalista para a realização do autocuidado de pessoas com IC durante a COVID-19.

## Método

### Delineamento do estudo

Estudo de abordagem qualitativa^14^, pautado na teoria de situação específica do autocuidado da insuficiência cardíaca, que traz como proposição teórica a influência do conhecimento, experiências, habilidade e compatibilidade com valores sobre o autocuidado de pessoas com IC^7^.

A elaboração e a execução da pesquisa foram norteadas pelas recomendações do *Consolidated Criteria for Reporting Qualitative Research* (COREQ): *a 32-item checklist for interviews and focus groups* ^15^, mediante *checklist* disponibilizado pela rede EQUATOR (https://www.equator-network.org/).

### Cenário da pesquisa

O estudo foi realizado com pessoas vinculadas à instituição hospitalar pública terciária do Sistema Único de Saúde (SUS), que presta atendimento ambulatorial e de internação hospitalar em cardiologia, de uma capital da região centro-oeste do país. As entrevistas ocorreram após a hospitalização em decorrência da descompensação da doença, utilizando como estratégia a consulta por telenfermagem^16^ ^), (^ ^17^.

### Período

As entrevistas foram realizadas entre novembro de 2020 e janeiro de 2021.

### Participantes do estudo e critérios de seleção

Participaram do estudo pessoas com idade igual ou superior a 18 anos, diagnosticadas com IC, sendo a causa da hospitalização registrada em prontuário. Os participantes foram selecionados de amostragem por variedade de tipos, definidos pelo critério da homogeneidade fundamental, cuja característica chave para a pesquisa era ter pelo menos uma readmissão hospitalar em até seis meses, por descompensação da IC^18^.

A busca dos pacientes foi feita pela listagem de internações mensais por especialidade de atendimento e motivo da internação de todos os que haviam sido readmitidos em até seis meses após a alta hospitalar.

Inicialmente foram selecionados 48 pacientes, desses, 22 não puderam ser contatados, pois estavam com registros desatualizados. A partir de uma busca ativa realizada por ligação telefônica, foram contatados 26 pacientes e realizadas nove exclusões: recusa na participação por debilidade física (2), óbito (1), não atender aos critérios de elegibilidade (1), não obter resposta em posterior contato telefônico (5). Um paciente foi considerado perda após o início da entrevista por queda na conexão da internet, sem sucesso numa nova conexão para continuidade da entrevista, mesmo após três tentativas, em dias alternados. Foram incluídos 16 participantes.

Os critérios de exclusão foram pessoas que apresentaram Acidente Vascular Cerebral (AVC) com sequelas cognitivas, gestantes, pacientes com IC de etiologia congênita, pacientes submetidos a cirurgia de grande porte três meses antes da coleta de dados, surdez e limitação cognitiva que dificultasse a comunicação.

### Coleta de dados

Após estabelecida a relação de pacientes elegíveis para o estudo, foi enviada uma carta-convite por meio da plataforma de mensagens instantâneas *WhatsApp* ^®^ reforçando o objetivo do estudo, apresentação da pesquisadora e razões para desenvolvimento do estudo.

Em virtude da restrição social gerada pela pandemia da COVID-19, todos os contatos com os participantes ocorreram pelo aplicativo de mensagens do próprio paciente ou familiar, e via ligação telefônica. A coleta de dados foi realizada por uma pesquisadora enfermeira com formação e experiência na condução de estudos qualitativos, em um único momento, em data e horário indicados pelo participante. Foi esclarecido sobre a priorização da entrevista por meio de vídeo chamada, porém por escolha de alguns participantes (N=6), a entrevista foi realizada por ligação de áudio.

A leitura do Termo de Consentimento Livre e Esclarecido foi gravada na íntegra, assim como toda a entrevista após o aceite, com recurso do aparelho móvel e disponíveis pelo sistema operacional iOS para *iPhone* ^®^. As entrevistas tiveram a duração média de 34 minutos (18 minutos - mínimo e 60 minutos - máxima).

O instrumento de coleta de dados consistiu de um roteiro semiestruturado que permitisse ao participante expressar sobre sua vivência com a doença, as medidas de autocuidado adotadas no dia a dia, as percepções sobre sintomas e condutas frente a sinais de descompensação, bem como informações que porventura tivessem recebido na internação sobre autocuidado.

As 5 questões foram elaboradas de acordo com a teoria que fundamenta o estudo^7^ ^), (^ ^8^ ^), (^ ^9^: 1) Você pode me falar como é viver com IC?; 2) Conte o que você tem feito no seu dia a dia para cuidar de si mesmo e da IC; 3) Quando você percebe que algo não está bem, quais atitudes que você toma que considera que possam colaborar para resolver o problema?; 4) O que você considera que pode ter contribuído para a ocorrência de mais de uma internação durante esse ano?; 5) Você tem algum exemplo de informações que recebeu de como cuidar melhor de si mesmo e de sua doença durante a internação?

Dados sociodemográficos e clínicos foram coletados dos prontuários e conferidos com os participantes no início da entrevista.

Após a coleta de dados devido ao período pandêmico, que gerou dificuldades de acesso ao serviço de saúde, muitas dúvidas e questionamentos foram suscitadas pelos participantes sobre as condutas adequadas no manejo da doença e questões inerentes ao serviço de saúde. Assim, devido ao compromisso ético da pesquisadora responsável pela coleta de dados, ao final da entrevista, foram fornecidas as informações solicitadas.

### Análise dos dados

Realizou-se a técnica de análise de conteúdo, modalidade temática^19^. O procedimento analítico por trilhas foi ancorado no referencial teórico da Teoria da Situação Específica do Autocuidado na Insuficiência Cardíaca^8^. A análise de conteúdo consiste em um conjunto de técnicas e procedimentos sistemáticos para compreensão do conteúdo das entrevistas buscando inferir conhecimento relevante. A leitura flutuante da entrevista após transcrição foi seguida de uma leitura minuciosa com o estabelecimento das unidades de significado, subcategorias e, por fim, as categorias que representassem o refinamento dos resultados^19^.

A análise temática proposta foi alcançada na leitura em profundidade e, para garantir o princípio da plausibilidade dos dados coletados e na interpretação dos resultados da pesquisa, a análise foi realizada por duas pesquisadoras e os caminhos de identificação das categorias foram discutidos buscando-se consenso sobre os temas identificados^18^.

Para sistematizar o processo de análise e realizar o gerenciamento do material textual, os dados foram processados com auxílio do *software* ATLAS.ti^®^ versão 9.0. As falas transcritas em documento do *Microsoft Word* foram direcionadas para o *software* para a criação das unidades de sentidos ou códigos, encontrados em quantidade expressiva (N=370). O recurso tecnológico promoveu maior rigor científico no agrupamento dos códigos, formando as subcategorias, das quais emergiram os temas.

Durante o processo as subcategorias e os temas foram analisados à luz da Teoria de Situação Específica do Autocuidado da IC, a fim de identificar se eles representavam as dimensões descritas nos elementos autônomos e consultivos da teoria.

Os participantes foram identificados com o código P seguido do número elegido a ele por ordem da entrevista (P1, P2, P3, (...), P16).

### Aspectos éticos

O estudo seguiu os preceitos éticos da pesquisa que envolve seres humanos, segundo a Resolução 466/2012 do Conselho Nacional de Saúde, sendo aprovado pela Instituição participante sob número de parecer 4.161.021, CAEE Nº 10225519.8.0000.5078, versão 3.

Após as entrevistas, conforme compromisso ético da pesquisa, enviou-se aos participantes um folheto informativo, elaborado pelos pesquisadores sobre autocuidado na IC, baseado nas etapas da Teoria da Situação Específica do Autocuidado da IC e pressupostos da Teoria de Médio Alcance das Doenças Crônicas^7^ ^), (^ ^8^ ^), (^ ^9^ ^), (^ ^20^.

## Resultados

Conforme a caracterização dos participantes do estudo (N=16), a maioria foi do sexo masculino (N=13), com idade entre 37 e 85 anos, especialmente acima de 65 anos (N=9), estado civil casado (N=10), com tempo de diagnóstico da doença maior do que 5 anos (N=12). A classificação da IC foi coletada da evolução médica registrada em prontuário. Foi predominante o registro do perfil clínico hemodinâmico com prevalência do Perfil B (N=10).

O perfil dos participantes refletiu elevado comprometimento cardíaco, com sintomatologia evidente e sinais de edema e congestão, além de comorbidades associadas, como a hipertensão arterial, alteração renal e/ou diabetes.

Considerando que o critério de inclusão do estudo foi ter sido submetido a mais de uma hospitalização, observou-se um número considerável de reospitalizações no período analisado. As internações autodeclaradas variaram entre duas a oito internações, sendo que oito participantes relataram três internações durante o ano e cinco haviam sido submetidos a mais de quatro internações, em intervalo de seis meses.

Ficou evidente por meio dos relatos que a instituição hospitalar de fato é a referência para o tratamento da doença. Contudo, as mudanças nos atendimentos devido à pandemia e as consequências sofridas por essa população refletem as barreiras impostas pelo serviço de saúde.

A leitura em profundidade e a análise do material derivado das entrevistas geraram duas categorias temáticas que se aproximam do processo de tomada de decisão naturalista (TDN) para a realização do autocuidado^7^ ^), (^ ^8^ ^), (^ ^9^, ressaltando as barreiras e limitações identificadas nas falas, conforme exemplificado na Figura 1.

**Figura 1:** Categorias e subcategorias da entrevista qualitativa do estudo “Autocuidado de pessoas com Insuficiência Cardíaca: a importância da telenfermagem na pandemia da COVID-19”. Brasil, 2023

Na Figura 2, são apresentadas as categorias e os aspectos de análise.

**Figura 2:** Aspectos de análise das categorias *Autocuidado como processo de tomada de decisão* e *Fatores que influenciam a tomada de decisão*. Brasil, 2023

### Autocuidado como processo de tomada de decisão

Nesta categoria estão os aspectos que influenciam as decisões de autocuidado no dia a dia dos participantes; conhecimento, experiência, habilidade e valores^7^ ^), (^ ^8^ ^), (^ ^9^. Todos esses aspectos foram identificados de forma bastante deficiente e em níveis muito abaixo do esperado, dado ao tempo de diagnóstico relatado. Dessa categoria surgiram duas subcategorias: *A entrevista como escuta terapêutica* e *O vínculo virtual como apoio para o autocuidado*.

Na subcategoria *A entrevista como escuta terapêutica* destacam-se estratos de fala que evidenciam a relevância do momento da entrevista por meio de relatos de problemas familiares, expressão dos sentimentos presentes na vivência com a IC, como sentimento de impotência, expressão da fé, preocupações e angústias:

*[...] gostava de trabalhar [...] sinto muita falta (emocionado) [...] sou muito católico também, e isso me ajuda muito [...]* (P1).

*[...] minha esposa faleceu tem pouco tempo e nós temos uma criança de três anos, está muito difícil, porque não posso trabalhar [...] tenho muitos problemas ainda* (emocionado) *[...]* (P9).

*[...] a minha vida tem sido muito difícil para mim, muito angustiante [...] eu procuro o jeito de rir, viver as coisas na graça, por que eu vivo com Deus [...] fica tão doendo, fica tão ruim, sabe? É muito difícil, é muito difícil, eu não sei nem o que eu faço sinceramente [...] eu pensava em controlar com alimento, né? [...]* (P11).

*[...] mas esse ano mesmo foi o pior ano da minha vida, de janeiro para cá [...] eu fui internado 6 vezes* (P12).

*[...] Quando o coração falha eu não faço nada, aí passa, aí eu ando, tento esquecer aquilo [...] o médico falou pra mim que um pouco é da cabeça, porque eu fico incutido com isso, então tento esquecer [...]* (P4).

*É cansativo demais. E você fica preocupado que você não sabe se você tá fazendo a coisa certa ou tá fazendo a coisa errada [...]* (P13).

*Trechos de fala foram destacados na subcategoria o vínculo virtual como apoio para o autocuidado*, os quais expressam solicitações de ajuda quanto a questões inerentes à doença e/ou procedimentos burocráticos pontuais. Percebeu-se, durante a entrevista, necessidades importantes de ajustes para controle da doença, que são imprescindíveis, e a falta de profissional que pudesse fornecer tal suporte. Observa-se que a coleta de dados alcançou a configuração de consulta de enfermagem e repercutiu as deficiências do período de internação para o aprendizado das pessoas com IC. Ademais, nota-se a relevância da telenfermagem no acompanhamento dos pacientes em ambiente domiciliar:

*[...] é verdadeiro ou eles tão enganando a gente* (referindo-se aos valores da Pressão Arterial do aparelho portátil)*? (...) Mas, é difícil. Eu queria ganhar ele* (referindo-se à medicação)*. Como é que eu faço pra mim ganhar ele?* (P8).

*Eu queria procurar assim se tem perigo de conforme a hora eu cair e morrer uma hora dessas, eu cair e eu morrer por causa dessa doença* (P10).

*Eu vou agendar seu telefone direitinho, e se eu tiver alguma dúvida, eu posso te perguntar?* (P12).

*[...] agora no sentido do coração quando eu passo mal [...] eu vou mesmo para o hospital [...] eu nem mexo com o diurético que eu já sei que não adianta, só no hospital [...] você sabe como me ajudar a conseguir laudo médico pra eu aposentar?* (P13).

*[...] minha esposa também tem problema do coração, tem dias a pressão dela está muito alta você pode falar com ela?* (passando telefone para a esposa) (P15).

### Fatores que influenciam a tomada de decisão

Os estratos de fala da categoria *Fatores que influenciam a tomada de decisão* representam os fatores ressaltados na teoria de situação específica do autocuidado da IC^8^: fatores pessoais, como as condições socioeconômicas, culturais, espirituais, letramento em saúde, presença de crenças fatalistas; fator problema, como a presença de mais de uma comorbidade, relatada por quase a totalidade dos participantes do estudo (N=13); e fatores ambientais, que estão relacionados ao apoio recebido, tanto de profissionais quanto familiares, para realização do autocuidado em seu ambiente familiar. Compreende também o suporte emocional para modular o estresse presente no gerenciamento da doença e na maneira como a pessoa lida com as emoções:

*[...] eu não estava passando bem, entendeu?* Aí ela não deu muito moral *pra mim* (médica) *[...] a emergência estava lotada, não tinha vaga [...] com essa pandemia me pediram para ficar em casa, mas aqui em casa eu estava passando muito mal* (P5).

*[...] tem dias que eu não durmo, passo a noite toda orando [...] eu fico muito mal, fico muito mal mesmo, só a cama pra melhorar. Eu fico um dia, dois, três, até quatro dias fico deitado pra melhorar* (P7).

*[...] hoje eu faço quase nada em pé, tem que fazer tudo sentada [...] eu cuido de mim mesma [...] (P8).*

*A minha esposa que lembra dos remédios, eu não dou conta* (P10).

*[...]* eu passo mal à noite, às vezes, eu ligo para ele (médico), ou minha mulher, e ele orienta. Ele é um anjo de Deus para mim [...] eu também tenho muita fé nos remédios caseiros (P12).

*Tem os programas* (cita nome do hospital*), mas por causa da pandemia eles foram suspensos. Eu estou esperando eles voltarem, como a reabilitação cardiovascular [...] vocês me deram uma atenção muito legal [...]* (P13).

*Quando eu era do programa* (participava de outro estudo desenvolvido no hospital)*, eu ligava pra secretária* (do médico) *agora não sou mais, eu não ligo mais [...] minha filha é enfermeira, então mesmo que eu não acho que não tem que ir, ela me faz ir* (para hospital) (P16).

## Discussão

Percebe-se, pelos estratos de fala dos participantes, um acompanhamento ambulatorial pouco expressivo, voltado especialmente ao pronto-atendimento em situações de maior complexidade. Os depoimentos convergem para o abandono principalmente das atividades físicas, realizadas no ambulatório de fisioterapia da instituição e/ou de forma espontânea em áreas públicas de lazer, dificuldades de atendimento devido à lotação das unidades de emergência, demora nas consultas de retorno pós alta e no ambulatório de cardiologia.

Quando questionados, a maioria dos participantes não tinha contato com profissionais de saúde para sanar dúvidas e orientar condutas do dia a dia, nem tampouco recebido orientação de como cuidar melhor de si durante a hospitalização. Quando as orientações eram realizadas, elas estavam mais relacionadas à rotina do serviço de nutrição oferecido a todos os pacientes, sem individualização das necessidades. Entretanto, mediante relatos de gratidão pela assistência recebida e a relação com a equipe de saúde, a presença do profissional médico foi enfatizada, sendo o enfermeiro - assim como outros profissionais da equipe multiprofissional - pouco evidenciado nas falas.

Um estudo multicêntrico testou programas de gestão da IC em instituições públicas e privadas no Brasil e alertou quanto à lacuna na educação ao paciente e cuidador, e à subutilização de monitoramento do paciente (visitas domiciliares e/ou telemonitoramento) em ambos tipos de serviços^21^. A baixa eficácia das informações fornecidas para redução de resultados negativos no tratamento da IC foi relacionada tanto ao tratamento farmacológico como ao não farmacológico.

São evidentes, também, lacunas de conhecimento acerca da patologia, do processo da doença, bem como das medidas não farmacológicas importantes na adoção de comportamentos de autocuidado que favoreçam melhor a qualidade de vida. Quando existentes, percebem-se relatos incipientes de tentativas de atitudes de autocuidado como controle do sódio, redução de ingesta de líquidos, realização de atividades físicas frequentes e monitoramento dos sintomas de descompensação. O tratamento farmacológico foi o mais comumente mencionado, porém com falhas na adesão.

Muitos participantes relataram dúvidas acerca de ajustes na alimentação e medicação e na busca por respostas acerca do estado clínico para sanar medos e inquietações. Outros expressaram dúvidas sobre procedimentos burocráticos na instituição hospitalar de referência, demostrando desejo de maior vínculo com os profissionais.

Na vigência de doenças crônicas, o autocuidado é essencial para a manutenção da saúde, requerendo um conjunto de comportamentos que levam à promoção da saúde e gestão da doença^20^. Diretrizes da IC enfatizam a importância da educação na adesão ao tratamento pelo paciente, nas mudanças do estilo de vida, no monitoramento dos sintomas e na resposta adequada a possíveis sinais de descompensação da doença^2^ ^), (^ ^22^.

A Teoria da Situação Específica do Autocuidado da IC define o autocuidado como um processo de tomada de decisão naturalista, sendo dividido em etapas lineares a serem percorridas pela pessoa com IC: 1) Manutenção do autocuidado, o qual envolve ajustes no estilo de vida, 2) A percepção dos sintomas, inclusos nessa etapa o monitoramento e processo de leitura corporal, e 3) O gerenciamento ou manejo do autocuidado, com aquisição de habilidades e adoção de condutas na vigência de alterações^7^ ^), (^ ^8^ ^), (^ ^9^.

Cada etapa envolve elementos consultivos, onde a pessoa recebe ajuda e apoio acerca do que fazer e desenvolve autonomia a partir da aquisição de conhecimento e confiança para cuidar de si^7^ ^), (^ ^8^ ^), (^ ^9^. À luz da teoria de situação específica do autocuidado da IC, as pessoas tomam decisões de forma automática, incompleta, contextual e raramente seguem um modelo. Entretanto, tais decisões podem ser influenciadas por diversos fatores, como: apoio emocional, suporte recebido, experiência, conhecimento, culturais, valores, entre outros^9^.

Deve ser considerado, ainda, o importante papel do cuidador informal, como o suporte profissional que possibilita a continuidade do cuidado e evita reospitalizações como única forma de resolução do problema^23^ ^), (^ ^24^ ^), (^ ^25^ ^), (^ ^26^.

A partir da pandemia da COVID-19, a teleconsulta de enfermagem foi regulamentada pela resolução COFEN nº 634/2020, a qual “autoriza e normatiza a teleconsulta de enfermagem mediante consultas, esclarecimentos, encaminhamentos e orientações com uso de meios tecnológicos”^27^. Logo, enfermeiros devem usar seus conhecimentos, habilidades e demais recursos disponíveis para atender às pessoas de forma mais abrangente e efetiva^28^ ^), (^ ^29^.

O autocuidado envolve múltiplos fatores que são suscetíveis de intervenção. É um processo passível de influências que pode favorecer melhores escolhas e resultar em melhores desfechos^30^. Desde a década de 80, a pesquisa sobre a tomada de decisão naturalista busca respostas sobre como as pessoas tomam decisões no mundo real^31^.

O número restrito de relatos, em nosso estudo, sobre informações recebidas durante a internação e o pouco conhecimento sobre os fatores que influenciam na descompensação da doença dificultam o processo de realização do autocuidado e tomada de decisão. Os participantes não demonstram estarem preparados para a tomada de decisão necessária em situações de descompensação.

Segundo a teoria de TDN, as pessoas fazem escolhas a partir de componentes racionais e/ou intuitivos, assim, ao realizar suas escolhas, raramente as fazem de forma analítica e sistemática. Ou seja, pessoas tomam decisões por diversos motivos, mas existe um consenso de que as experiências vivenciadas desencadeiam escolhas rápidas diante de situações críticas de modo que se comprometam com um curso de ação, sem necessariamente comparar as demais alternativas existentes^31^ ^), (^ ^32^.

Em muitas entrevistas, percebeu-se a expressão de desabafos de experiências ao se receber o diagnóstico, a menção aos valores pessoais, o impacto das mudanças no estilo de vida, a falta de motivação para cuidarem de si, relatos de situações traumáticas e que influenciam na adesão ao tratamento e na aquisição de autonomia para o manejo do autocuidado. Evidências essas que dizem da necessidade dos participantes em serem ouvidos em suas angústias e anseios.

Os participantes do estudo parecem possuir conhecimento insuficiente, mesmo com tempo considerável de diagnóstico; quando o possuem, poucos relatam segurança para adoção de atitudes coerentes. Exemplo disso é a negativa quanto à autonomia para ingestão de diurético a mais na vigência de edema.

Segundo os conceitos descritos na teoria de médio alcance do autocuidado das doenças crônicas, a motivação pode ser intrínseca e extrínseca, sendo que a primeira surge do desejo de aprender e executar tarefas que geram prazer ou satisfação, e a extrínseca surge da compreensão da necessidade de melhora da saúde, ou, ainda, visando a agradar outras pessoas, como familiares^20^.

Muitos comportamentos de autocuidado que surgem da motivação extrínseca evoluem para a motivação intrínseca, configurando-se num estilo de vida prazeroso para a pessoa e com maiores chances de sucesso^20^. Tal explicação se relaciona com o entendimento da tomada de decisão a partir de combinações de padrões apreendidos, fator puramente intuitivo. Caso a pessoa realize uma correspondência clara sobre suas escolhas e os resultados obtidos, poderá realizar ações mais coerentes com sua condição de saúde e necessidades, envolvendo escolhas mais assertivas a partir do raciocínio frente às diferentes situações existentes^31^ ^), (^ ^33^.

As crenças e valores culturais também ficaram bastante evidenciados, como o alto nível de dependência do cônjuge, influências da religiosidade, crenças populares em medicações caseiras e demais condutas que podem ser preocupantes, contradizendo recomendações baseadas em evidências científicas.

Devido à falta de acompanhamento, ficou evidente a sobrecarga emocional dos participantes deste estudo. As necessidades explícitas ou subentendidas em muitos momentos de fala refletem a carência em se ter alguém que tivesse, de fato, interesse por ouvir seus depoimentos, histórias de vida e do cotidiano, frustrações, dificuldades, angústias, anseios, ou, ainda, alguém que pudesse fornecer um suporte psicológico no enfrentamento da doença.

Condições psicológicas podem contribuir de forma mais negativa para a piora da doença em comparação às causas fisiológicas, devido ao impacto na vida das pessoas, ao acompanhamento ausente dessa abordagem e/ou gestão inadequada dos sintomas^34^.

Nesse sentido, o pesquisador ocupa um lugar importante nas relações humanas, de compreensão subjetiva da experiência do outro, independentemente das informações fazerem sentido para o próprio pesquisador^35^. Na pesquisa qualitativa, o participante e o pesquisador são partes interligadas na investigação, em que a compreensão do fenômeno depende de um movimento empático, sensível e singular^14^. Os significados das conversas depende do contexto em que elas ocorrem^15^.

Atribuímos a profundidade dos temas que surgiram durante a entrevista à abordagem qualitativa que, embora tenha sido utilizada em um roteiro de entrevista com questões semiestruturadas, permitiu esse momento de empatia com a pesquisadora, escuta terapêutica e ambiente propício para que os participantes expusessem sua realidade e sentimentos.

A escuta terapêutica é compreendida como uma estratégia essencial para entendimento do outro, capaz de definir uma ordem de prioridade de assistência a partir das necessidades individuais expressas pelas pessoas, sendo capaz de proporcionar aos profissionais de saúde maneiras de melhorar o desempenho das pessoas em suas tomadas de decisão^31^ ^), (^ ^36^.

O estudo fenomenológico sobre o distanciamento social durante a pandemia ressalta que a impossibilidade de interagir presencialmente exerceu impacto profundo nas relações sociais^37^, ao passo que “a interação com outra pessoa pode nutrir uma sensação de conforto e esperança ou, pelo contrário, uma sensação de desconforto e vulnerabilidade”^37^. É preciso considerar o contexto das interações interpessoais compartilhado pelo outro e o “estar no mundo” já existente da outra pessoa, em que somos convidados a participar, possibilitando a profundidade de interpretação e o processo de ajuda.

Ainda muito desconhecidas, essas experiências virtuais devem ser analisadas a fim de trazer maior clareza das dimensões desse novo tempo, na expectativa de trazer respostas positivas acerca de uma nova maneira de prestar assistência ao outro. Nossos dados fortalecem, ainda, a compreensão sobre a necessidade de intervenções abrangentes e uma assistência de enfermagem mais acessível às pessoas em seu ambiente domiciliar.

A confiança, mencionada anteriormente como elemento fundamental para o manejo do autocuidado, reflete a autoeficácia das pessoas, ou seja, a capacidade de tomada de decisão coerente mesmo na vigência de barreiras e dificuldades, não fazendo parte do autocuidado em si, mas sendo imprescindível em todas as etapas^7^ ^), (^ ^8^ ^), (^ ^9^. Fica evidente a necessidade de apoio e estímulo para com os participantes do estudo.

A análise das entrevistas leva-nos a perceber que, na vigência de algum evento inesperado (extrínseco; como a pandemia ou outra situação estressante do cotidiano, ou intrínseco; como na descompensação da IC e exacerbação de sintomas), é importante que a pessoa seja capaz de realizar o processo de tomada de decisão para o autocuidado, nesse sentido, a telenfermagem pode ser um recurso colaborativo na gestão desses eventos, no fortalecimento de vínculo estabelecido previamente, ou na possibilidade de estabelecer esse vínculo em período pós-hospitalização. Para representar essa interrelação, foi elaborado um diagrama, representado na Figura 3.

Na Política Nacional da Atenção Básica (PNAB), o vínculo “consiste na construção de relações de afetividade e de confiança entre o usuário e o trabalhador da saúde, permitindo o aprofundamento do processo de corresponsabilização pela saúde, construído ao longo do tempo, além de carregar, em si, um potencial terapêutico”^38^.

**Figura 3:** Diagrama representativo da relação entre o vínculo com as pessoas com IC, a influência da telenfermagem e o processo de tomada de decisão naturalista para o autocuidado em situações de eventos inesperados. Brasil, 2023

Por meio da telenfermagem, o estabelecimento do vínculo pode ser iniciado na aquisição de informações que possibilitem intervenções mesmo à distância, orientações de estilo de vida e esclarecimento de dúvidas, de modo a prevenir a readmissão precoce^39^.

A afirmação verificada em um estudo realizado por enfermeiras capacitadas em tele-*coach*, na Alemanha, que atendeu pessoas com IC no período pós-alta durante a pandemia da COVID-19, demonstrou que o grupo acompanhado por telemonitoramento reconheceu os sintomas de descompensação da doença e apreendeu o momento de procura da assistência médica, evitando situações de maior gravidade^40^.

Em nosso estudo, de forma semelhante, a telenfermagem representou uma ferramenta importante nesse contexto de crise sanitária mundial, ao promover empatia, respeito, escuta ativa e um cuidado terapêutico individualizado e integral aos pacientes com IC.

O estudo contribui para o avanço da ciência ao demonstrar que a utilização da telenfermagem é capaz de ultrapassar barreiras importantes impostas entre pessoas com IC e os serviços de saúde. Contudo, o uso de teorias da prática pode instrumentalizar e direcionar a assistência dos enfermeiros e demais membros da equipe de saúde na elucidação de dúvidas e no apoio ao alcance das etapas do autocuidado. Mais estudos de abordagem qualitativa são necessários para promover novas evidências dessas relações.

Este estudo teve como limitações a diversidade dos fatores sociodemográficos e clínicos dos participantes e as dificuldades apresentadas por alguns participantes no uso de recursos eletrônicos de comunicação, em especial das mídias de vídeo.

## Conclusão

O estudo mostrou, à luz da Teoria da Situação Específica do Autocuidado da Insuficiência Cardíaca e da Teoria de Médio Alcance do Autocuidado de Doenças Crônicas, a existência de lacunas importantes que comprometem a tomada de decisão de pessoas com IC, as quais impactam significativamente na aquisição de habilidades para o gerenciamento adequado do autocuidado.

Ao se considerar o período pandêmico, essa população sofreu consequências negativas da falta do acompanhamento necessário para a gestão da doença. Em vista disso, a telenfermagem é um recurso importante para estabelecimento de vínculo com o paciente após período de hospitalização.

A presença do enfermeiro foi pouco percebida pelos relatos, trazendo um alerta sobre a falta da presença ativa do profissional enquanto educador, o que resulta na deficiência do conhecimento e na compreensão dessas pessoas no contexto dos serviços de saúde. Essa percepção ficou clara nas solicitações realizadas pelos participantes do estudo com as dúvidas apresentadas e valorização desse momento de entrevista.

Ficou evidente a necessidade de continuidade da assistência e estabelecimento de uma rede de apoio à pessoa após a hospitalização. A entrevista configurou-se em um momento terapêutico integrante da telenfermagem, haja vista as barreiras impostas pela pandemia da COVID-19.

Além disso, ressaltou-se a importância do apoio emocional à pessoa com IC, aliada ao tratamento convencional, visando ao melhor enfrentamento da doença. Entretanto, é necessária uma reflexão sobre o quão efetivo é o acompanhamento dessa população em todos os contextos, mesmo fora da ótica do período pandêmico.

# Translation: El autocuidado de personas con insuficiencia cardíaca: la importancia de la teleenfermería en la pandemia de COVID-19*

**DOI:** 10.1590/1518-8345.6975.4226

**Authors:** Micaelle Costa Gondim, Ricardo Costa da Silva, Ana Karoline Barbosa da Silva, Flaviana Vely Mendonça Vieira, Janaína Valadares Guimarães, Karina Machado Siqueira, Agueda Maria Ruiz Zimmer Cavalcante

**Affiliations:** 1 Universidade Federal de Goiás, Faculdade de Enfermagem, Goiânia, GO, Brasil; 2 Becario(a) de la Coordenação de Aperfeiçoamento de Pessoal de Nível Superior (CAPES), Brasil; 3 Universidade Estadual de Goiás, Ceres, GO, Brasil

**Keywords:** Enfermedad Crónica, Automanejo, Investigación Cualitativa, Insuficiencia Cardíaca, COVID-9, Telemonitorización

## Abstract

**Objetivo:**

describir factores de influencia de la teleenfermería en la toma de decisión naturalista sobre el autocuidado de personas con insuficiencia cardíaca durante la pandemia de COVID-19.

**Método:**

estudio descriptivo de enfoque cualitativo, con 16 participantes entrevistados tras la hospitalización por la descompensación de la insuficiencia cardíaca. Los datos se recopilaron mediante tecnología de audio y vídeo y se sometieron a análisis de contenido, guiado por la Teoría de Situación Específica del Autocuidado de la Insuficiencia Cardíaca.

**Resultados:**

dos categorías temáticas surgieron del análisis: *Autocuidado como proceso de toma de decisión* y *Factores que influyen en la toma de decisión*, las cuales describen a la teleenfermería como recurso de apoyo a la persona con insuficiencia cardíaca, durante modificaciones de atención en el período pandémico.

**Conclusión:**

fue posible comprender la relación entre la teleenfermería y el establecimiento de vínculo, de manera a estimular la toma de decisión naturalista por la persona con insuficiencia cardíaca en su cotidianidad de forma coherente, impulsando mejores resultados en salud.

## Destacados

**(1)** Diversas barreras comprometen la toma de decisiones de personas con insuficiencia cardíaca.

**(2)** Las teorías de enfermería pueden dirigir actitudes coherentes de autocuidado.

**(3)** Es necesaria la continuidad de la asistencia y la red de apoyo tras la hospitalización.

**(4)** El apoyo emocional a la persona con insuficiencia cardíaca debe estar aliado al tratamiento convencional.

**(5)** El vínculo por medio de la teleenfermería proporciona desenlaces positivos en salud.

## Introducción

De los diversos aspectos en los que podríamos analizar el contexto de la crisis sanitaria mundial, tomamos como objeto de observación para este estudio a las personas con insuficiencia cardíaca (IC), ya que pertenecen al grupo de enfermedades crónicas, considerado condiciones de mayor vulnerabilidad para el nuevo tipo de Coronavirus, el *Severe Acute Respiratory Syndrome Coronavirus* 2 - SARS-COV 2, causante de la enfermedad COVID-19 a consecuencia del síndrome respiratorio agudo^1^.

Como enfermedad crónica, la IC resulta de importantes modificaciones fisiológicas, estructurales y funcionales, asociada a la presencia de multimorbilidades, elevadas tasas de rehospitalización^2^ y coexistencia de múltiples síntomas emocionales que contribuyen significativamente a los eventos de descompensación cardiovascular^3^.

Sin embargo, debido a la pandemia de COVID-19, hubo un cambio importante en los servicios de salud a nivel mundial, tanto a nivel de ambulatorio como a hospitalario, ocasionando una reducción en el seguimiento a esta población y, consecuentemente, en el número de internaciones hospitalarias^4^.

En el año 2020, hubo más de 114 mil internaciones por IC en todo el país, siendo 11.548 admisiones (y readmisiones) en la región Centro-Oeste, lugar de este estudio^5^. Un número expresivo, sin embargo, con una reducción del 15% de las internaciones en comparación con el año anterior^4^. Esta falta de seguimiento puede influir en la realización del autocuidado llevando a la descompensación cardíaca^6^.

La Teoría de la Situación Específica del Autocuidado de la IC instrumentaliza a los enfermeros para una asistencia de enfermería basada en el incentivo de cambios importantes en el estilo de vida, en la percepción y monitoreo de los síntomas y en el alcance de autonomía para la realización del autocuidado por medio de un proceso de toma de decisión naturalista (TDN)^7^ ^), (^ ^8^ ^), (^ ^9^.

Las personas con enfermedades crónicas presentan demandas importantes en el curso de la enfermedad y necesitan ajustes en la rutina para el autocuidado^10^. El vínculo comprende la responsabilidad entre el equipo de salud y el individuo en la promoción de la salud, prevención de agravios por medio de la comunicación efectiva, construcción de confianza y relaciones de afectividad^11^.

Cuando este vínculo se mantiene, por ejemplo, con el telemonitoreo , refuerza el potencial de esta herramienta tecnológica para prestar cuidados a distancia, facilitar el acceso al profesional de salud, favorecer la práctica clínica, el cuidado individualizado, el esclarecimiento de dudas, miedos y ansiedades, favoreciendo el conocimiento de diferentes realidades de las personas asistidas^12^ ^), (^ ^13^.

Consideramos importante el abordaje del tema, pues más allá de las particularidades que involucran el enfrentamiento de la pandemia durante el período de restricciones sociales, existen lagunas importantes en la literatura sobre el autocuidado de personas con IC fundamentado en teorías de la práctica que aborden limitaciones y barreras encontradas en el experimentar de la enfermedad. Mediante las experiencias relatadas tras la hospitalización, la investigación buscó describir los factores de influencia de la teleenfermería en la toma de decisión naturalista para la realización del autocuidado de personas con IC durante la COVID-19.

## Método

### Diseño del estudio

Estudio de enfoque cualitativo^14^, basado en la teoría de situación específica del autocuidado de la insuficiencia cardíaca, que trae como proposición teórica la influencia del conocimiento, experiencias, habilidad y compatibilidad con valores sobre el autocuidado de personas con IC^7^.

La elaboración y ejecución de la investigación fueron guiadas por las recomendaciones del *Consolidated Criteria for Reporting Qualitative Research* (COREQ): una lista de verificación de 32 ítems para entrevistas y grupos focales^15^, mediante una lista de verificación disponible por la red EQUATOR (https://www.equator-network.org/).

### Escenario de la investigación

El estudio se realizó con personas vinculadas a una institución hospitalaria pública terciaria del Sistema Único de Salud (SUS), que brinda atención ambulatoria y de internación hospitalaria en cardiología, en una capital de la región centro-oeste del país. Las entrevistas se llevaron a cabo después de la hospitalización debido a la descompensación de la enfermedad, utilizando como estrategia la consulta por teleenfermería^16^ ^), (^ ^17^.

### Período

Las entrevistas se realizaron entre noviembre de 2020 y enero de 2021.

### Participantes del estudio y criterios de selección

Participaron del estudio personas de 18 años o más, diagnosticadas con IC, siendo la causa de la hospitalización registrada en el expediente. Los participantes fueron seleccionados de una muestra por variedad de tipos, definidos por el criterio de homogeneidad fundamental, cuya característica clave para la investigación era tener al menos una readmisión hospitalaria en hasta seis meses, por descompensación de la IC^18^.

La búsqueda de los pacientes se realizó por el listado de internaciones mensuales por especialidad de atención y motivo de la internación de todos los que habían sido readmitidos en hasta seis meses después del alta hospitalaria.

Inicialmente se seleccionaron 48 pacientes, y de estos, 22 no pudieron ser contactados, ya que tenían registros desactualizados. Por medio de una búsqueda activa realizada por llamada telefónica, se contactaron a 26 pacientes y se realizaron nueve exclusiones: rechazo a participar por debilidad física (2), fallecimiento (1), no cumplir con los criterios de elegibilidad (1), no obtener respuesta en un contacto telefónico posterior (5). Un paciente fue considerado pérdida después del inicio de la entrevista por caída de la conexión de internet, sin éxito en una nueva conexión para continuar la entrevista, incluso después de tres intentos, en días alternos. Se incluyeron 16 participantes.

Los criterios de exclusión fueron personas que presentaron Accidente Cerebrovascular (ACV) con secuelas cognitivas, embarazadas, pacientes con IC de etiología congénita, pacientes sometidos a cirugía de gran porte tres meses antes de la recolección de datos, sordera y limitación cognitiva que dificultara la comunicación.

### Recolección de datos

Tras establecer la relación de pacientes elegibles para el estudio, se envió una carta de invitación por medio de la plataforma de mensajes instantáneos *WhatsApp* ^®^ reforzando el objetivo del estudio, presentación de la investigadora y razones para el desarrollo del estudio.

Debido a la restricción social generada por la pandemia de COVID-19, todos los contactos con los participantes ocurrieron por la aplicación de mensajes del propio paciente o familiar, y mediante llamada telefónica. La recolección de datos fue realizada por una investigadora enfermera con formación y experiencia en la conducción de estudios cualitativos, en un único momento, en fecha y hora indicados por el participante. Se aclaró sobre la priorización de la entrevista mediante videollamada, sin embargo, por elección de algunos participantes (N=6), la entrevista se realizó por llamada de audio.

La lectura del consentimiento informado fue grabada en la íntegra, así como toda la entrevista tras la aceptación, con recurso del dispositivo móvil y disponibles por el sistema operativo iOS para *iPhone* ^®^. Las entrevistas tuvieron una duración media de 34 minutos (18 minutos - mínimo y 60 minutos - máximo).

El instrumento de recolección de datos consistió en un guion semiestructurado que permitió a los participantes expresar su experiencia con la enfermedad, las medidas de autocuidado adoptadas en su día a día, las percepciones sobre síntomas y conductas ante señales de descompensación, así como información que posiblemente habían recibido durante la hospitalización sobre autocuidado.

Las 5 preguntas se elaboraron de acuerdo con la teoría que fundamenta el estudio^7^ ^), (^ ^8^ ^), (^ ^9^: 1) ¿Podría hablar sobre cómo es vivir con IC?; 2) Cuénteme qué ha hecho en su día a día para cuidarse a sí mismo y su IC; 3) Cuando nota que algo no va bien, ¿qué acciones toma que considera que pueden ayudar a resolver el problema?; 4) ¿Qué considera que pudo haber contribuido a más de una hospitalización durante este año?; 5) ¿Tiene algún ejemplo de información que recibió sobre cómo cuidarse mejor a sí mismo y su enfermedad durante la hospitalización?

Los datos sociodemográficos y clínicos se recolectaron de los expedientes médicos y se verificaron con los participantes al inicio de la entrevista.

Tras la recolección de datos, debido al periodo pandémico, que generó dificultades de acceso al servicio de salud, surgieron muchas dudas y preguntas por parte de los participantes sobre las conductas adecuadas en el manejo de la enfermedad y cuestiones inherentes al servicio de salud. Por lo tanto, debido al compromiso ético de la investigadora responsable de la recolección de datos, al final de la entrevista, se proporcionó la información solicitada.

### Análisis de los datos

Se utilizó la técnica de análisis de contenido, modalidad temática^19^. El procedimiento analítico por rastreo estuvo anclado en el marco teórico de la Teoría de la Situación Específica del Autocuidado en la Insuficiencia Cardíaca^8^. El análisis de contenido consiste en un conjunto de técnicas y procedimientos sistemáticos para la comprensión del contenido de las entrevistas buscando inferir conocimientos relevantes. La lectura flotante de la entrevista tras la transcripción fue seguida por una lectura minuciosa con el establecimiento de unidades de significado, subcategorías y, finalmente, las categorías que representaban el refinamiento de los resultados^19^.

El análisis temático propuesto se logró en la lectura en profundidad y para asegurar el principio de plausibilidad de los datos recolectados y en la interpretación de los resultados de la investigación, el análisis fue realizado por dos investigadoras y los caminos de identificación de las categorías se discutieron buscando consenso sobre los temas identificados^18^.

Para sistematizar el proceso de análisis y gestionar el material textual, los datos se procesaron con ayuda del *software* ATLAS.ti^®^ versión 9.0. Las declaraciones transcritas en documento de *Microsoft Word* se dirigieron al *software* para la creación de unidades de sentidos o códigos, encontrados en cantidad expresiva (N=370). Este recurso tecnológico promovió un mayor rigor científico en el agrupamiento de los códigos formando las subcategorías, de las cuales emergieron los temas.

Durante este proceso, las subcategorías y los temas se analizaron a la luz de la Teoría de Situación Específica del Autocuidado de la IC, con el fin de identificar si representaban las dimensiones descritas en los elementos autónomos y consultivos de la teoría.

Los participantes se identificaron con el código P seguido del número asignado a él por orden de entrevista (P1, P2, P3, (...), P16).

### Aspectos éticos

El estudio siguió los preceptos éticos de la investigación que involucra seres humanos, según la Resolución 466/2012 del Consejo Nacional de Salud, siendo aprobado por la Institución participante bajo el número de dictamen 4.161.021, CAEE Nº 10225519.8.0000.5078, versión 3.

Después de las entrevistas, como compromiso ético de la investigación, se enviaron a los participantes un folleto informativo, elaborado por los investigadores sobre autocuidado en la IC, basado en las etapas de la teoría de situación específica del autocuidado de la IC y presupuestos de la teoría de alcance medio de las enfermedades crónicas^7^ ^), (^ ^8^ ^), (^ ^9^ ^), (^ ^20^.

## Resultados

Según la caracterización de los participantes del estudio (N=16), la mayoría fueron hombres (N=13), con edades entre 37 y 85 años, especialmente mayores de 65 años (N=9), estado civil casado (N=10), con tiempo de diagnóstico de la enfermedad de más de 5 años (N=12). La clasificación de la IC se recopiló de la evolución médica registrada en el expediente. Predominó el registro del perfil clínico hemodinámico con prevalencia del Perfil B (N=10).

El perfil de los participantes reflejó un alto compromiso cardíaco, con sintomatología evidente y signos de edema y congestión, además de comorbilidades asociadas como la hipertensión arterial, alteración renal y/o diabetes.

Considerando que el criterio de inclusión del estudio fue haber sido sometido a más de una hospitalización, se observó un número considerable de rehospitalizaciones en el período analizado. Las internaciones autodeclaradas variaron entre dos a ocho hospitalizaciones, siendo que ocho participantes reportaron tres hospitalizaciones durante el año y cinco habían sido sometidos a más de cuatro, en un intervalo de seis meses.

Quedó evidente mediante los relatos que la institución hospitalaria es de hecho la referencia para el tratamiento de la enfermedad. Sin embargo, los cambios en los servicios de salud debido a la pandemia y las consecuencias sufridas por esta población reflejan las barreras impuestas por el servicio de salud.

La lectura en profundidad y el análisis del material derivado de las entrevistas generaron dos categorías temáticas que se acercan al proceso de TDN para la realización del autocuidado^7^ ^), (^ ^8^ ^), (^ ^9^, resaltando las barreras y limitaciones identificadas en las declaraciones, como se ejemplifica en la Figura 1.

**Figura 1:** Categorías y subcategorías de la entrevista cualitativa del estudio “Autocuidado de personas con Insuficiencia Cardíaca: la importancia de la teleenfermería en la pandemia de COVID-19”. Brasil, 2023

La Figura 2 presenta las categorías y aspectos de análisis.

**Figura 2:** Aspectos de análisis de las categorías *Autocuidado como proceso de toma de decisión y Factores que influyen en la toma de decisión*. Brasil, 2023

### Autocuidado como proceso de toma de decisión

En esta categoría están los aspectos que influyen en las decisiones de autocuidado en el día a día de los participantes; conocimiento, experiencia, habilidad y valores^7^ ^), (^ ^8^ ^), (^ ^9^. Todos estos aspectos fueron identificados de forma bastante deficiente y en niveles muy por debajo de lo esperado, dado al tiempo de diagnóstico relatado. De esta categoría surgieron dos subcategorías: *La entrevista como escucha terapéutica* y *El vínculo virtual como apoyo para el autocuidado*.

En la subcategoría *La entrevista como escucha terapéutica* se destacan extractos de habla que evidencian la relevancia del momento de la entrevista mediante relatos de problemas familiares, expresión de los sentimientos presentes en la vivencia con la IC; como sentimiento de impotencia, expresión de la fe, preocupaciones y angustias:

*[...] me gustaba trabajar [...] extraño mucho (emocionado) [...] soy muy católico también, y eso me ayuda mucho [...]* (P1).

*[...] mi esposa falleció hace poco tiempo y tenemos un niño de tres años, está muy difícil, porque no puedo trabajar [...] tengo muchos problemas aún (emocionado) [...]* (P9).

*[...] mi vida ha sido muy difícil para mí, muy angustiosa [...] intento encontrar la manera de reír, vivir las cosas en gracia, porque vivo con Dios [...] duele tanto, se siente tan mal, ¿sabes? Es muy difícil, es muy difícil, sinceramente no sé qué hacer [...] pensaba en controlarlo con comida, ¿verdad? [...]* (P11).

*[...] pero este año ha sido el peor año de mi vida, desde enero para acá [...] he sido hospitalizado 6 veces* (P12).

*[...] Cuando falla el corazón no hago nada, luego pasa, entonces camino, trato de olvidarlo [...] el médico me dijo que un poco es en la cabeza, porque me obsesiono con eso, entonces trato de olvidarlo [...]* (P4).

*Es demasiado agotador. Y te preocupas porque no sabes si estás haciendo lo correcto o estás haciendo lo incorrecto [...]* (P13).

Extractos de habla fueron destacados en la subcategoría el *vínculo virtual como apoyo para el autocuidado*, los cuales expresan solicitudes de ayuda en cuanto a cuestiones inherentes a la enfermedad y/o procedimientos burocráticos puntuales. Se percibió durante la entrevista necesidades importantes de ajustes para el control de la enfermedad, que son imprescindibles, y la falta de ese profesional que pudiera proporcionar tal soporte. Se observa que la recolección de datos alcanzó la configuración de consulta de enfermería y repercutió las deficiencias del periodo de internación para el aprendizaje de las personas con IC. Además, se nota la relevancia de la teleenfermería en el seguimiento de los pacientes en ambiente domiciliario:

*[...] ¿es verdad o nos están engañando* (refiriéndose a los valores de la Presión Arterial del aparato portátil)*? (...) Pero, es difícil. Yo quería ganarlo* (refiriéndose a la medicación)*. ¿Cómo hago para conseguirlo?* (P8).

*Quería averiguar así si hay peligro de que en cualquier momento me caiga y muera, me caiga y muera por esta enfermedad* (P10).

*Voy a agendar tu teléfono correctamente, ¿y si tengo alguna duda, puedo preguntarte?* (P12).

*[...] ahora en sentido del corazón cuando me siento mal [...] voy directo al hospital [...] ni me meto con el diurético porque ya sé que no sirve, solo en el hospital [...] ¿sabes cómo ayudarme a conseguir un informe médico para jubilarme?* (P13).

*[...] mi esposa también tiene problemas del corazón, hay días que su presión está muy alta ¿puedes hablar con ella?* (pasando el teléfono a la esposa) (P15).

### Factores que influyen en la toma de decisión

Los extractos de las declaraciones de la categoría *Factores que influyen en la toma de decisión* representan los factores resaltados en la teoría de situación específica del autocuidado de la IC^8^: *factores personales* como las condiciones socioeconómicas, culturales, espirituales, alfabetización en salud, presencia de creencias fatalistas; el *factor problema* como la presencia de más de una comorbilidad, reportada por casi la totalidad de los participantes del estudio (N=13); y *factores ambientales* que están relacionados con el apoyo recibido, tanto de profesionales como familiares, para realizar el autocuidado en su entorno familiar. También comprende el soporte emocional para modular el estrés presente en el manejo de la enfermedad y en la forma en que la persona maneja las emociones:

*[...] no me sentía bien, ¿sabes? Entonces ella (la médica) no me prestó mucha atención [...] la emergencia estaba llena, no había espacio [...] con esta pandemia me pidieron quedarme en casa, pero aquí en casa me sentía muy mal* (P5).

*[...] hay días que no duermo, paso toda la noche rezando [...] me siento muy mal, realmente mal, solo la cama me mejora. Me quedo uno, dos, tres, hasta cuatro días acostado para mejorar* (P7).

*[...] hoy no hago casi nada de pie, tengo que hacer todo sentada [...] yo me cuido a mí misma [...] (P8).*

*Mi esposa es la que recuerda los medicamentos, yo no puedo* (P10).

*[...] a veces me siento mal por la noche, llamo a él* (médico)*, o mi esposa, y él me orienta. Él es un ángel de Dios para mí [...] también tengo mucha fe en los remedios caseros* (P12).

*Hay programas* (menciona el nombre del hospital)*, pero por la pandemia fueron suspendidos. Estoy esperando que vuelvan, como la rehabilitación cardiovascular [...] ustedes me han dado mucha atención [...]* (P13).

*Cuando estaba en el programa* (participaba de otro estudio desarrollado en el hospital)*, llamaba a la secretaria* (del médico) *ahora ya no, ya no llamo más [...] mi hija es enfermera, entonces incluso si creo que no es necesario, ella me hace ir* (al hospital) (P16).

## Discusión

Se percibe, por medio de los extractos de las declaraciones de los participantes, un seguimiento ambulatorio poco significativo, orientado especialmente a la atención de urgencia en situaciones de mayor complejidad. Los testimonios convergen hacia el abandono, principalmente de las actividades físicas, realizadas en el ambulatorio de fisioterapia de la institución y/o de forma espontánea en áreas públicas de ocio, dificultades de atención debido a la saturación de las unidades de emergencia, demora en las consultas de retorno post alta y en el ambulatorio de cardiología.

Cuando se les preguntó, la mayoría de los participantes no tenía contacto con profesionales de la salud para aclarar dudas y orientar conductas cotidianas, ni tampoco había recibido orientación sobre cómo cuidarse mejor durante la hospitalización. Cuando se realizaban las orientaciones, estaban más relacionadas con la rutina del servicio de nutrición ofrecido a todos los pacientes, sin individualización de las necesidades. Sin embargo, mediante relatos de gratitud por la asistencia recibida y la relación con el equipo de salud, se enfatizó la presencia del profesional médico, siendo el enfermero, así como otros profesionales del equipo multiprofesional, poco destacados en las declaraciones.

Un estudio multicéntrico probó programas de gestión de la IC en instituciones públicas y privadas en Brasil y alertó sobre la brecha en la educación del paciente y cuidador, y la subutilización del monitoreo del paciente (visitas domiciliarias y/o telemonitoreo) en ambos tipos de servicios^21^. La baja eficacia de la información proporcionada para reducir los resultados negativos en el tratamiento de la IC, se relacionaron tanto con el tratamiento farmacológico como no farmacológico.

También son evidentes las lagunas de conocimiento acerca de la patología, el proceso de la enfermedad, así como de las medidas no farmacológicas importantes en la adopción de comportamientos de autocuidado que favorezcan una mejor calidad de vida. Cuando existen, se perciben relatos incipientes de intentos de actitudes de autocuidado como el control del sodio, reducción de la ingesta de líquidos, realización de actividades físicas frecuentes y monitoreo de los síntomas de descompensación. El tratamiento farmacológico fue el más comúnmente mencionado, pero con fallos en la adherencia.

Muchos participantes reportaron dudas sobre ajustes en la alimentación y medicación y en la búsqueda de respuestas acerca del estado clínico para aliviar miedos e inquietudes. Otros expresaron dudas sobre procedimientos burocráticos en la institución hospitalaria de referencia demostrando el deseo de un mayor vínculo con los profesionales.

En la presencia de enfermedades crónicas, el autocuidado es esencial para el mantenimiento de la salud, requiriendo un conjunto de comportamientos que llevan a la promoción de la salud y gestión de la enfermedad^20^. Las directrices de la IC enfatizan la importancia de la educación en la adherencia al tratamiento por parte del paciente, en los cambios de estilo de vida, el monitoreo de los síntomas y la respuesta adecuada a posibles señales de descompensación de la enfermedad^2^ ^), (^ ^22^.

La teoría de la situación específica del autocuidado de la IC define el autocuidado como un proceso de toma de decisión naturalista, dividido en etapas lineales a ser recorridas por la persona con IC: 1) Mantenimiento del autocuidado, que involucra ajustes en el estilo de vida, 2) La percepción de los síntomas, incluido en esta etapa el monitoreo y proceso de lectura corporal, y 3) El manejo o gestión del autocuidado, con la adquisición de habilidades y adopción de conductas ante cambios^7^ ^), (^ ^8^ ^), (^ ^9^.

Cada etapa involucra elementos consultivos, donde la persona recibe ayuda y soporte sobre qué hacer y desarrolla autonomía a partir de la adquisición de conocimiento y confianza para cuidarse^7^ ^), (^ ^8^ ^), (^ ^9^. A la luz de la teoría de situación específica del autocuidado de la IC, las personas toman decisiones de manera automática, incompletas, contextual y raramente siguen un modelo. Sin embargo, pueden ser influenciadas por diversos factores, como: apoyo emocional, soporte recibido, experiencia, conocimiento, culturales, valores entre otros^9^.

También debe considerarse el importante papel del cuidador informal, así como el soporte profesional que posibilita la continuidad del cuidado y evita las rehospitalizaciones como única forma de resolución del problema^23^ ^), (^ ^24^ ^), (^ ^25^ ^), (^ ^26^.

Desde la pandemia de COVID-19, la teleconsulta de enfermería fue regulada por la resolución COFEN nº 634/2020, que “autoriza y normatiza la teleconsulta de enfermería mediante consultas, esclarecimientos, derivaciones y orientaciones con uso de medios tecnológicos”^27^. Por lo tanto, los enfermeros deben utilizar sus conocimientos, habilidades y demás recursos disponibles para atender a las personas de manera más amplia y efectiva^28^ ^), (^ ^29^.

El autocuidado involucra múltiples factores susceptibles de intervención. Es un proceso influenciable que puede favorecer mejores elecciones y resultar en mejores desenlaces^30^. Desde la década de los 80, la investigación sobre la toma de decisión naturalista busca respuestas sobre cómo las personas toman decisiones en el mundo real^31^.

El número limitado de relatos en nuestro estudio sobre información recibida durante la hospitalización y el escaso conocimiento sobre los factores que influyen en la descompensación de la enfermedad dificultan el proceso de realización del autocuidado y toma de decisión. Los participantes no parecen estar preparados para la toma de decisión necesaria en situaciones de descompensación.

Según la teoría de TDN, las personas hacen elecciones a partir de componentes racionales y/o intuitivos, y al realizar sus elecciones, raramente lo hacen de forma analítica y sistemática. Es decir, las personas toman decisiones por diversos motivos, pero hay un consenso de que las experiencias vividas desencadenan elecciones rápidas ante situaciones críticas de manera que se comprometen con un curso de acción, sin necesariamente comparar las demás alternativas existentes^31^ ^), (^ ^32^.

En muchas entrevistas se percibieron desahogos de experiencias al recibir el diagnóstico, mención a los valores personales, el impacto de los cambios en el estilo de vida, la falta de motivación para cuidarse, relatos de situaciones traumáticas que influyen en la adhesión al tratamiento y en la adquisición de autonomía para el manejo del autocuidado. Estas son evidencias de la necesidad de los participantes de ser escuchados en sus angustias y anhelos.

Los participantes del estudio parecen tener conocimiento insuficiente, incluso con un tiempo considerable desde el diagnóstico; cuando lo tienen, pocos informan seguridad para la adopción de actitudes coherentes. Un ejemplo de ello es la negativa sobre la autonomía para la ingesta de un diurético adicional en presencia de edema.

Según conceptos descritos en la teoría de medio alcance del autocuidado de las enfermedades crónicas, la motivación puede ser intrínseca y extrínseca, siendo la primera surgida del deseo de aprender y ejecutar tareas que generan placer o satisfacción, y la extrínseca surge de la comprensión de la necesidad de mejora de la salud, o incluso buscando agradar a otras personas, como familiares^20^.

Muchos comportamientos de autocuidado que surgen de la motivación extrínseca evolucionan hacia la motivación intrínseca, configurándose en un estilo de vida placentero para la persona y con mayores posibilidades de éxito^20^. Esta explicación se relaciona con el entendimiento de la toma de decisiones a partir de combinaciones de patrones aprendidos, factor puramente intuitivo. Si la persona realiza una correspondencia clara sobre sus elecciones y los resultados obtenidos, podrá realizar acciones más coherentes con su condición de salud y necesidades e involucrar elecciones más asertivas a partir del razonamiento frente a las diferentes situaciones existentes^31^ ^), (^ ^33^.

Las creencias y valores culturales también quedaron muy evidenciados, como el alto nivel de dependencia del cónyuge, influencias de la religiosidad, creencias populares en medicaciones caseras y demás conductas que pueden ser preocupantes, contradiciendo recomendaciones basadas en evidencias científicas.

Debido a la falta de seguimiento, quedó evidente la sobrecarga emocional de los participantes de este estudio. Las necesidades explícitas o subyacentes en muchos momentos de las declaraciones reflejan la carencia de tener a alguien que realmente tuviera interés en escuchar sus testimonios, historias de vida y del día a día, frustraciones, dificultades, angustias, anhelos, o incluso, alguien que pudiera ofrecer un soporte psicológico en el enfrentamiento de la enfermedad.

Las condiciones psicológicas pueden contribuir más negativamente al empeoramiento de la enfermedad que las causas fisiológicas, debido al impacto en la vida de las personas, al seguimiento ausente de este enfoque y/o a la gestión inadecuada de los síntomas^34^.

En este sentido, el investigador ocupa un lugar importante en las relaciones humanas de comprensión subjetiva de la experiencia del otro, independientemente de que la información tenga sentido para el propio investigador^35^. En la investigación cualitativa, el participante y el investigador son partes interconectadas en la investigación, donde la comprensión del fenómeno depende de un movimiento empático, sensible y singular^14^. Los significados de las conversaciones dependen del contexto en que ocurren^15^.

Atribuimos la profundidad de los temas que surgieron durante la entrevista al enfoque cualitativo que, aunque se utilizó un guion de entrevista con preguntas semiestructuradas, permitió este momento de empatía con la investigadora, escucha terapéutica y un ambiente propicio para que los participantes expusieran su realidad y sentimientos.

Esta escucha terapéutica se entiende como una estrategia esencial para la comprensión del otro, capaz de definir un orden de prioridad de asistencia a partir de las necesidades individuales, expresadas por las personas y capaz de informar a los profesionales de la salud sobre maneras de mejorar el desempeño de las personas en sus tomas de decisiones^31^ ^), (^ ^36^.

Un estudio fenomenológico sobre el distanciamiento social durante la pandemia resalta que la imposibilidad de interactuar presencialmente tuvo un impacto profundo en las relaciones sociales^37^, en el sentido de que “la interacción con otra persona puede nutrir una sensación de confort y esperanza o, por el contrario, una sensación de incomodidad y vulnerabilidad”^37^. Es necesario considerar el contexto de las interacciones interpersonales compartido por el otro y el “estar en el mundo” ya existente de la otra persona, en el que somos invitados a participar, y que posibilita la profundidad de interpretación y el proceso de ayuda.

Aún muy desconocidas, estas experiencias virtuales deben ser analizadas con el fin de traer mayor claridad sobre las dimensiones de este nuevo tiempo, con la expectativa de traer respuestas positivas de una nueva manera de prestar asistencia al otro. Nuestros datos fortalecen también la comprensión sobre la necesidad de intervenciones amplias y una asistencia de enfermería más accesible para las personas en su entorno domiciliario, donde surgen las mayores dudas y dificultades, el papel en el núcleo familiar y la identidad como ser social.

La confianza mencionada anteriormente, como elemento fundamental para el manejo del autocuidado, refleja la autoeficacia de las personas, es decir, la capacidad de tomar decisiones coherentes incluso en presencia de barreras y dificultades, no siendo parte del autocuidado en sí, pero imprescindible en todas las etapas^7^ ^), (^ ^8^ ^), (^ ^9^. Se hace evidente la necesidad de apoyo y estímulo para con los participantes del estudio.

El análisis de las entrevistas nos lleva a percibir que, en la presencia de algún evento inesperado (extrínseco; como la pandemia u otra situación estresante cotidiana, o intrínseco; como en la descompensación de la IC y exacerbación de síntomas) es importante que la persona sea capaz de realizar el proceso de toma de decisiones para el autocuidado, en este sentido la teleenfermería puede ser un recurso colaborativo en la gestión de estos eventos, en el fortalecimiento del vínculo establecido previamente, o en la posibilidad de establecer este vínculo en el período post-hospitalización. Para representar esta interrelación se elaboró un diagrama representado en la Figura 3.

**Figura 3:** Diagrama representativo de la relación entre el vínculo con las personas con insuficiencia cardíaca, la influencia de la teleenfermería y el proceso de toma de decisión naturalista para el autocuidado en situaciones de eventos inesperados. Brasil, 2023

En la Política Nacional de Atención Primaria (PNAB), el vínculo “consiste en la construcción de relaciones de afectividad y confianza entre el usuario y el trabajador de la salud, permitiendo la profundización del proceso de corresponsabilización por la salud, construido a lo largo del tiempo, además de llevar en sí un potencial terapéutico” (p. 21)^38^.

Por intermedio de la teleenfermería, el establecimiento del vínculo puede iniciarse en la adquisición de información que posibilite intervenciones incluso a distancia, orientaciones de estilo de vida y aclaración de dudas, de modo a prevenir la readmisión precoz^39^.

Esta afirmación se verificó en un estudio realizado por enfermeras capacitadas en *tele-coaching*, en Alemania, durante la pandemia de COVID-19, que atendió a personas con IC en el período post-alta, demostrando que el grupo acompañado por telemonitoramiento reconoció los síntomas de descompensación de la enfermedad y aprendió sobre el momento de buscar asistencia médica, evitando situaciones de mayor gravedad^40^.

De manera similar en nuestro estudio, la teleenfermería representó una herramienta importante en este contexto de crisis sanitaria mundial al promover empatía, respeto, escucha activa y un cuidado terapéutico individualizado e integral a los pacientes con IC.

El estudio contribuye al avance de la ciencia al demostrar que la utilización de la teleenfermería es capaz de superar barreras importantes impuestas entre las personas con IC y los servicios de salud. Sin embargo, el uso de teorías de la práctica puede instrumentalizar y dirigir la asistencia de los enfermeros y otros miembros del equipo de salud en la elucidación de dudas y en el apoyo para alcanzar las etapas del autocuidado. Se necesitan más estudios de enfoque cualitativo para promover nuevas evidencias de estas relaciones.

Este estudio tuvo como limitación la diversidad de factores sociodemográficos y clínicos de los participantes y las dificultades presentadas por algunos participantes en el uso de recursos electrónicos de comunicación, especialmente los medios de video.

## Conclusión

El estudio mostró, a la luz de la teoría de situación específica del autocuidado de la insuficiencia cardíaca y la teoría de alcance medio del autocuidado de enfermedades crónicas, la existencia de importantes brechas que comprometen la toma de decisiones de las personas con IC, las cuales impactan significativamente en la adquisición de habilidades para la gestión adecuada del autocuidado.

Al considerar el período pandémico, esta población sufrió consecuencias negativas por la falta del seguimiento necesario para la gestión de la enfermedad. En vista de ello, la teleenfermería es un recurso importante para el establecimiento de vínculo con el paciente después del período de hospitalización.

La presencia del enfermero fue poco percibida por los relatos, lanzando una alerta sobre la ausencia de la presencia activa del profesional como educador, lo que resulta en la deficiencia del conocimiento y comprensión de estas personas en el contexto de los servicios de salud. Esta percepción quedó clara en las solicitudes realizadas por los participantes del estudio con las dudas presentadas y la valorización de ese momento de la entrevista.

Quedó evidente la necesidad de continuidad de la asistencia y establecimiento de una red de apoyo a la persona después de la hospitalización. La entrevista se configuró en un momento terapéutico integrante de la teleenfermería, dadas las barreras impuestas por la pandemia de COVID-19.

Además, se resaltó la importancia del apoyo emocional a la persona con IC, aliado al tratamiento convencional, buscando un mejor enfrentamiento de la enfermedad. Sin embargo, es necesaria una reflexión sobre cuán efectivo es el seguimiento a esta población en todos los contextos, incluso fuera de la óptica del período pandémico.
